# Supplementary material for: Non-canonical Metatranscriptomic analysis of COVID-19 and Dengue reveals an expanded microbial and AMR landscape in COVID-19 mortality patients
Source: PLoS Pathog. 2025 Nov 19;21(11):e1013703. doi: 10.1371/journal.ppat.1013703 (PMC12629440; doi:10.1371/journal.ppat.1013703)
Supplement: S1 File — (DOCX) [file ppat.1013703.s001.docx]

**Non-canonical Metatranscriptomic analysis of COVID-19 and Dengue reveals an expanded microbial and AMR landscape in COVID-19 mortality patients**

Aanchal Yadav^1,3,6^, Raiyan Ali^1,6^, Priti Devi^1,3^, Pallawi Kumari^1,4^, Jyoti Soni^1,3^, Garima^1,3^, Bansidhar Tarai^5^, Sandeep Budhiraja^5^, Uzma Shamim^1,2,*^ , Rajesh Pandey^1,3,7,*^

^1^Division of Immunology and Infectious Disease Biology, INtegrative GENomics of HOst-PathogEn (INGEN-HOPE) laboratory, CSIR-Institute of Genomics and Integrative Biology (CSIR-IGIB), Mall Road, Delhi-110007, India.

^2^Ashoka University, Sonipat, Haryana-131029, India

^3^Academy of Scientific and Innovative Research (AcSIR), Ghaziabad-201002, India.

^4^Indraprastha Institute of Information Technology (IIIT), New Delhi-110020, India

^5^Max Super Speciality Hospital (A Unit of Devki Devi Foundation), Max Healthcare, Delhi 110017, India.

^6^Equal contribution

^*^Co-corresponding authors

^7^Lead contact

Contact Details:

**Rajesh Pandey, PhD**

Principal Scientist,

INtegrative GENomics of HOst-PathogEn (INGEN-HOPE) laboratory,

CSIR-Institute of Genomics and Integrative Biology (CSIR-IGIB),

North Campus, Near Jubilee Hall, Mall Road, Delhi-110007, India.

Contact: [rajeshp@igib.in](mailto:rajeshp@igib.in); [rajesh.p@igib.res.in](mailto:rajesh.p@igib.res.in); Tel.: 011-27002200 (Ext. 254)

**Running title:** Resistome and Microbiome Dynamics in COVID-19 and Dengue

**Supplementary File S1: Sensitivity Analysis Within the Dengue Cohort to Assess and Address Potential Sample Type Bias.**

While, our primary aim was to characterise the transcriptionally active microbes and resistome at the relevant site of infection for each disease: the respiratory tract for COVID-19 and the bloodstream for Dengue – we acknowledge that comparing clinical specimens from different body sites—nasopharyngeal swabs for COVID-19 and blood for Dengue—can introduce potential variability due to the site-specific microbiome differences. Although, the site specific sample collection was for the diseases studies, which are united by single stranded RNA viruses, causing infection.

Thus, to assess potential disease-specific microbial signatures independent of sample type, we performed a sensitivity analysis within the Dengue cohort, using an independent lab generated dataset. Towards this, we included a dengue-negative control group comprising 20 febrile patients who tested NS1 antigen-negative, and whose blood total RNA samples were processed and sequenced using the same protocol as for the 112 dengue-positive samples. Microbial taxa and antibiotic resistance genes derived from unmapped reads were compared between the groups, and statistical differences were assessed using PERMANOVA with 999 random permutations. Random permutations were controlled by stratifying the site origin type using strata argument within adonis2 function of vegan package in R, wherein we observed distinct clustering, suggesting that the observed TAMs and ARGs differences are attributable to disease status rather than the sampling site.

This analysis reinforces the biological relevance of the disease-specific microbial profiles, while we still acknowledge that between-disease comparisons may be influenced by differing sample origins.

1. TAMs abundance (Dengue positive and Dengue negative patients)


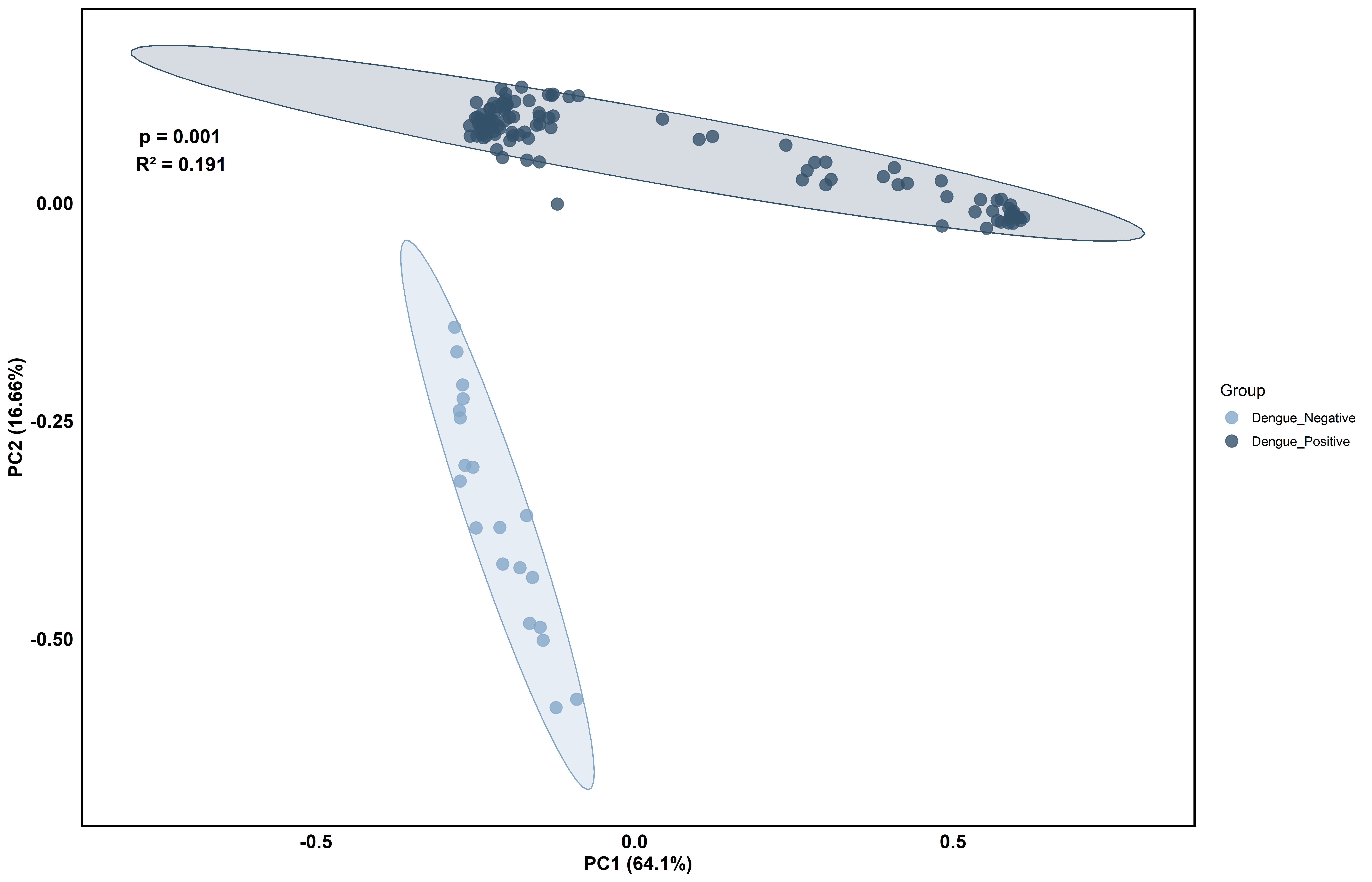


1. ARGs abundance (Dengue positive and Dengue negative patients)


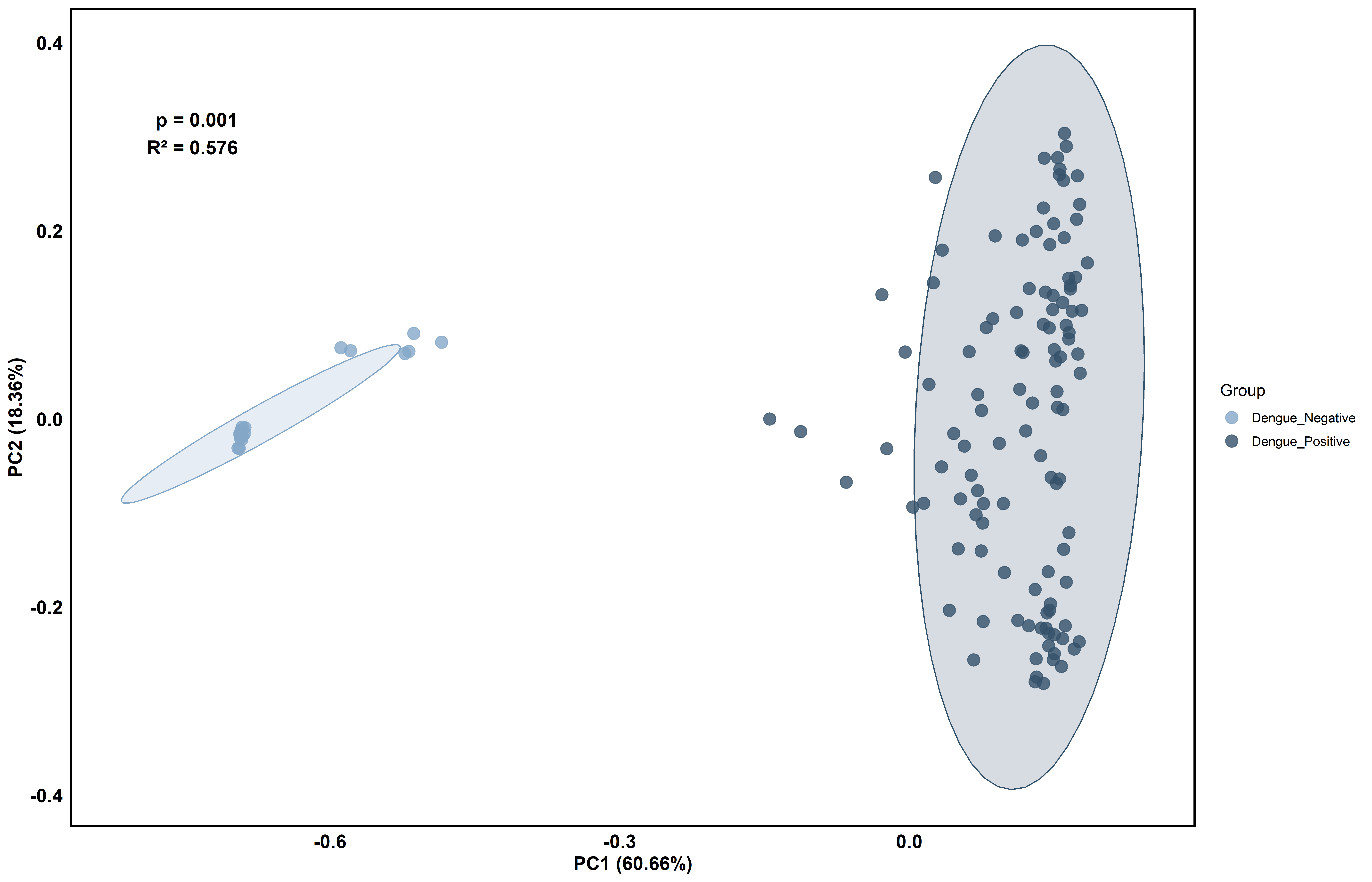


**Figure:** PCoA Plots representing distinct abundance clustering of (a) TAMs and (b) ARGs between dengue-positive and dengue-negative samples.
