# Supplementary material for: Non-canonical Metatranscriptomic analysis of COVID-19 and Dengue reveals an expanded microbial and AMR landscape in COVID-19 mortality patients
Source: PLoS Pathog. 2025 Nov 19;21(11):e1013703. doi: 10.1371/journal.ppat.1013703 (PMC12629440; doi:10.1371/journal.ppat.1013703)
Supplement: S2 File — (DOCX) [file ppat.1013703.s002.docx]

**Non-canonical Metatranscriptomic analysis of COVID-19 and Dengue reveals an expanded microbial and AMR landscape in COVID-19 mortality patients**

Aanchal Yadav^1,3,6^, Raiyan Ali^1,6^, Priti Devi^1,3^, Pallawi Kumari^1,4^, Jyoti Soni^1,3^, Garima^1,3^, Bansidhar Tarai^5^, Sandeep Budhiraja^5^, Uzma Shamim^1,2,*^ , Rajesh Pandey^1,3,7,*^

^1^Division of Immunology and Infectious Disease Biology, INtegrative GENomics of HOst-PathogEn (INGEN-HOPE) laboratory, CSIR-Institute of Genomics and Integrative Biology (CSIR-IGIB), Mall Road, Delhi-110007, India.

^2^Ashoka University, Sonipat, Haryana-131029, India

^3^Academy of Scientific and Innovative Research (AcSIR), Ghaziabad-201002, India.

^4^Indraprastha Institute of Information Technology (IIIT), New Delhi-110020, India

^5^Max Super Speciality Hospital (A Unit of Devki Devi Foundation), Max Healthcare, Delhi 110017, India.

^6^Equal contribution

^*^Co-corresponding authors

^7^Lead contact

Contact Details:

**Rajesh Pandey, PhD**

Principal Scientist,

INtegrative GENomics of HOst-PathogEn (INGEN-HOPE) laboratory,

CSIR-Institute of Genomics and Integrative Biology (CSIR-IGIB),

North Campus, Near Jubilee Hall, Mall Road, Delhi-110007, India.

Contact: [rajeshp@igib.in](mailto:rajeshp@igib.in); [rajesh.p@igib.res.in](mailto:rajesh.p@igib.res.in); Tel.: 011-27002200 (Ext. 254)

**Running title:** Resistome and Microbiome Dynamics in COVID-19 and Dengue

**Supplementary File S2: Alpha diversity (Chao1, Observed and Simpson indices) of ARGs and TAMs in COVID-19 and dengue infection.**

1. ARGs alpha diversity

i. ii.

**
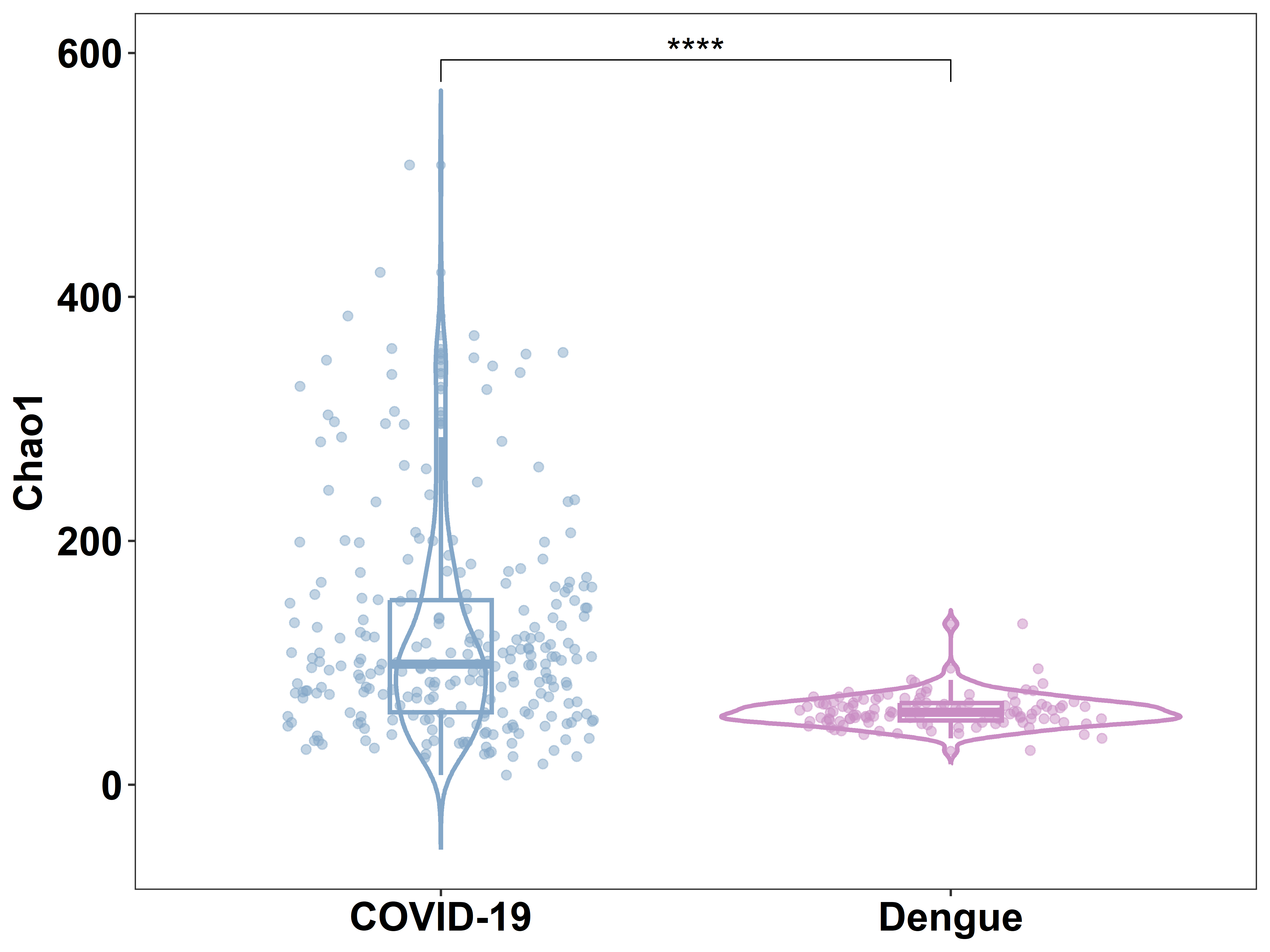
**
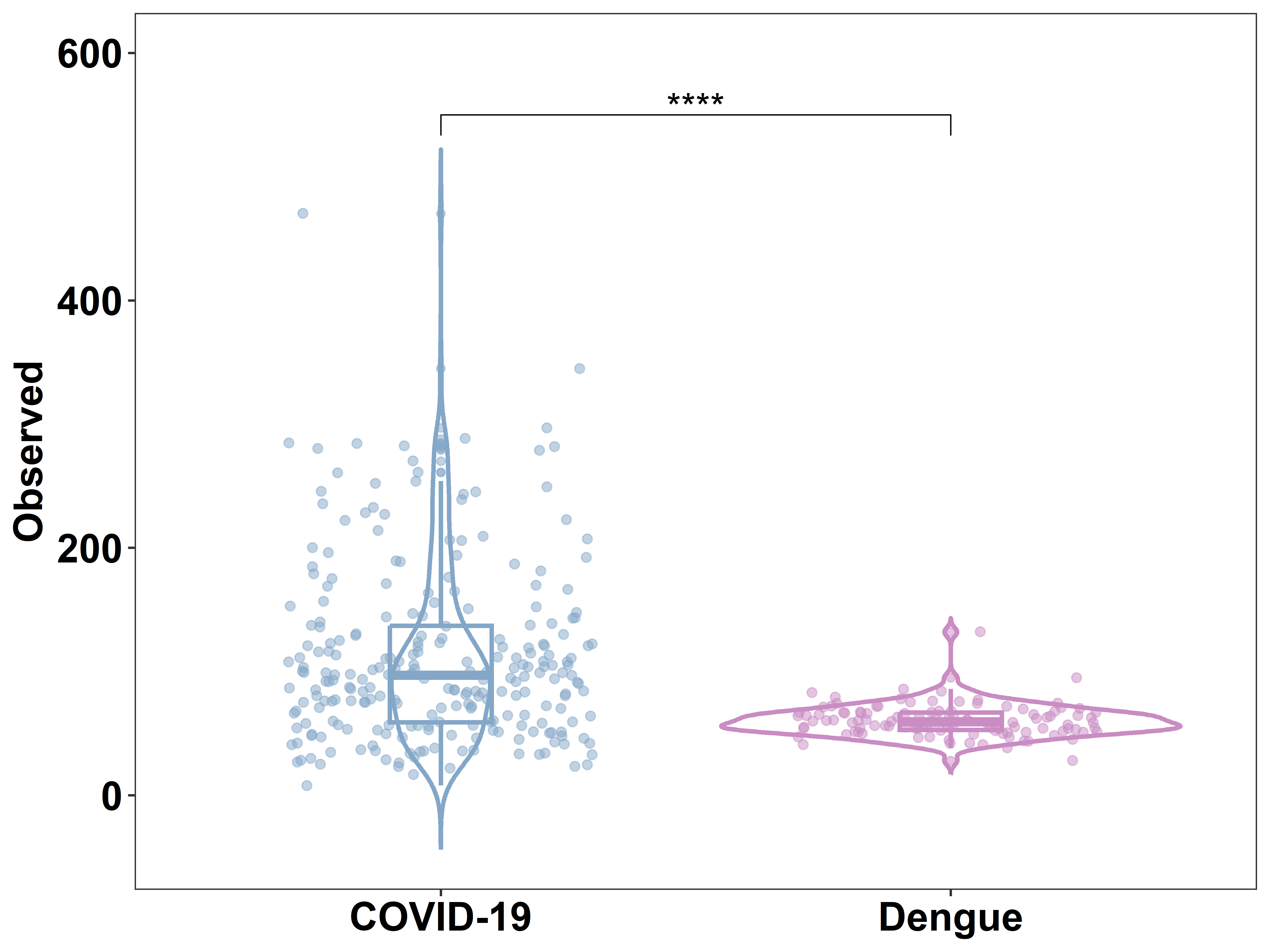


iii.


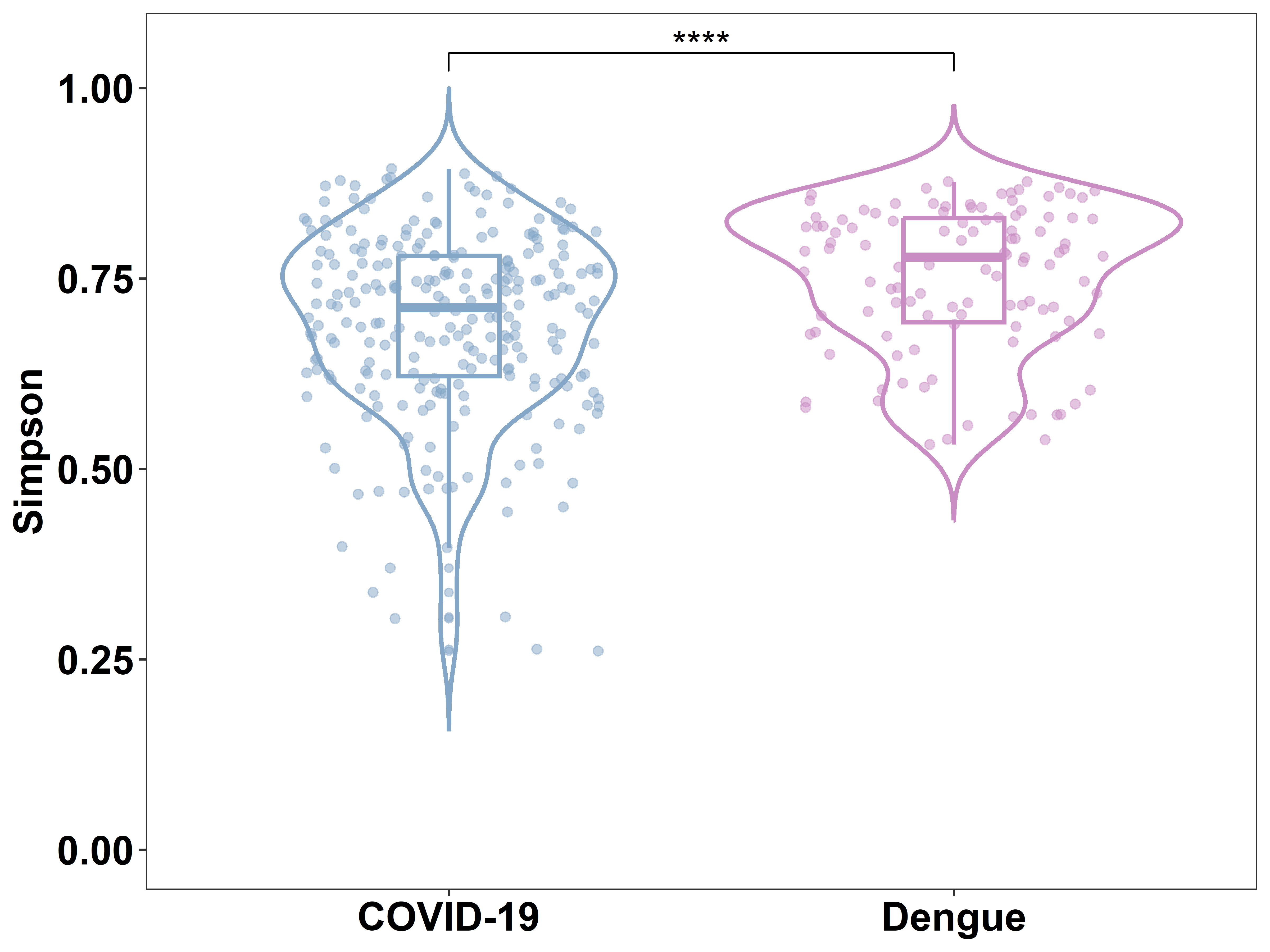


1. TAMs alpha diversity

i. ii.


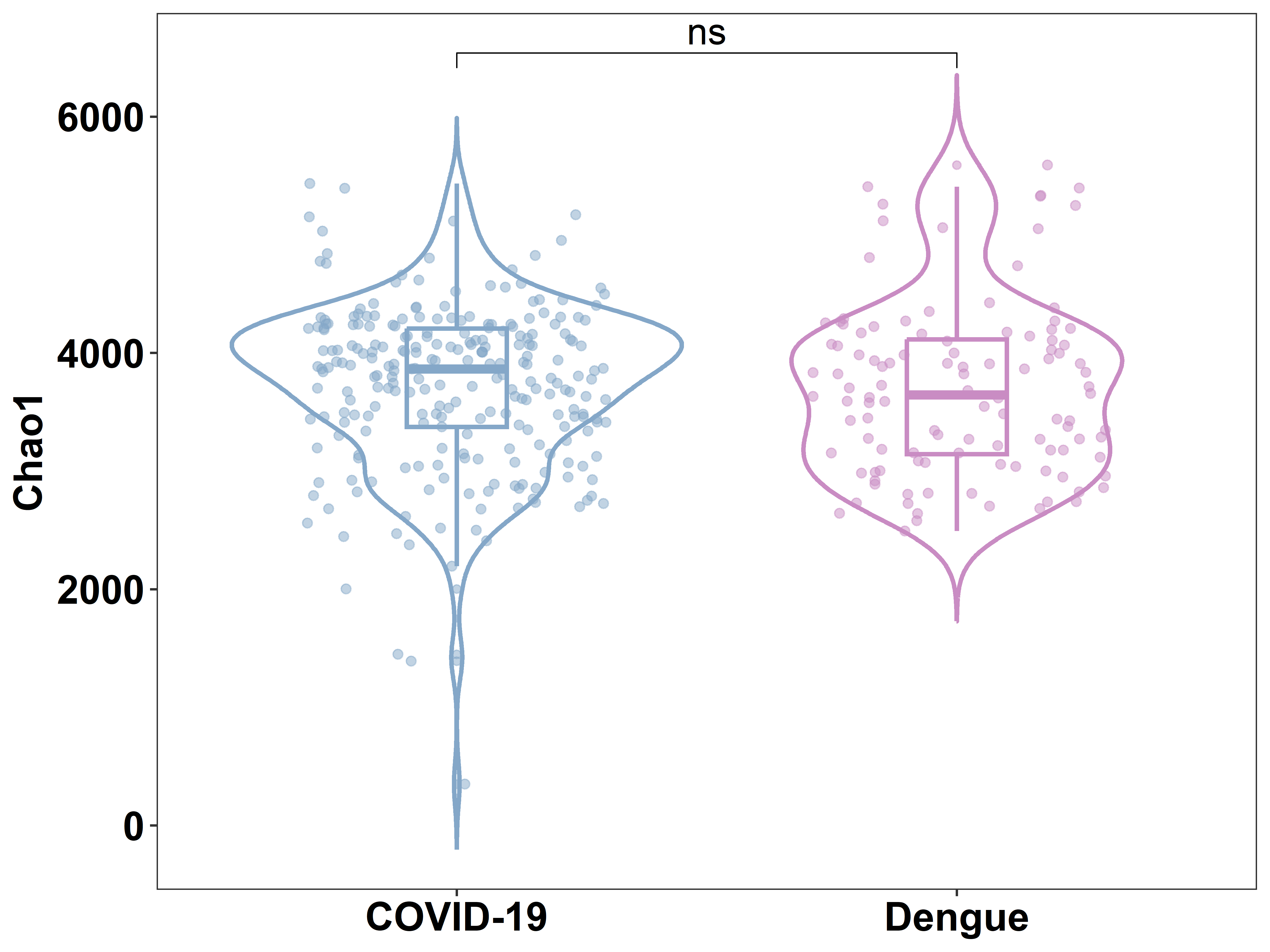

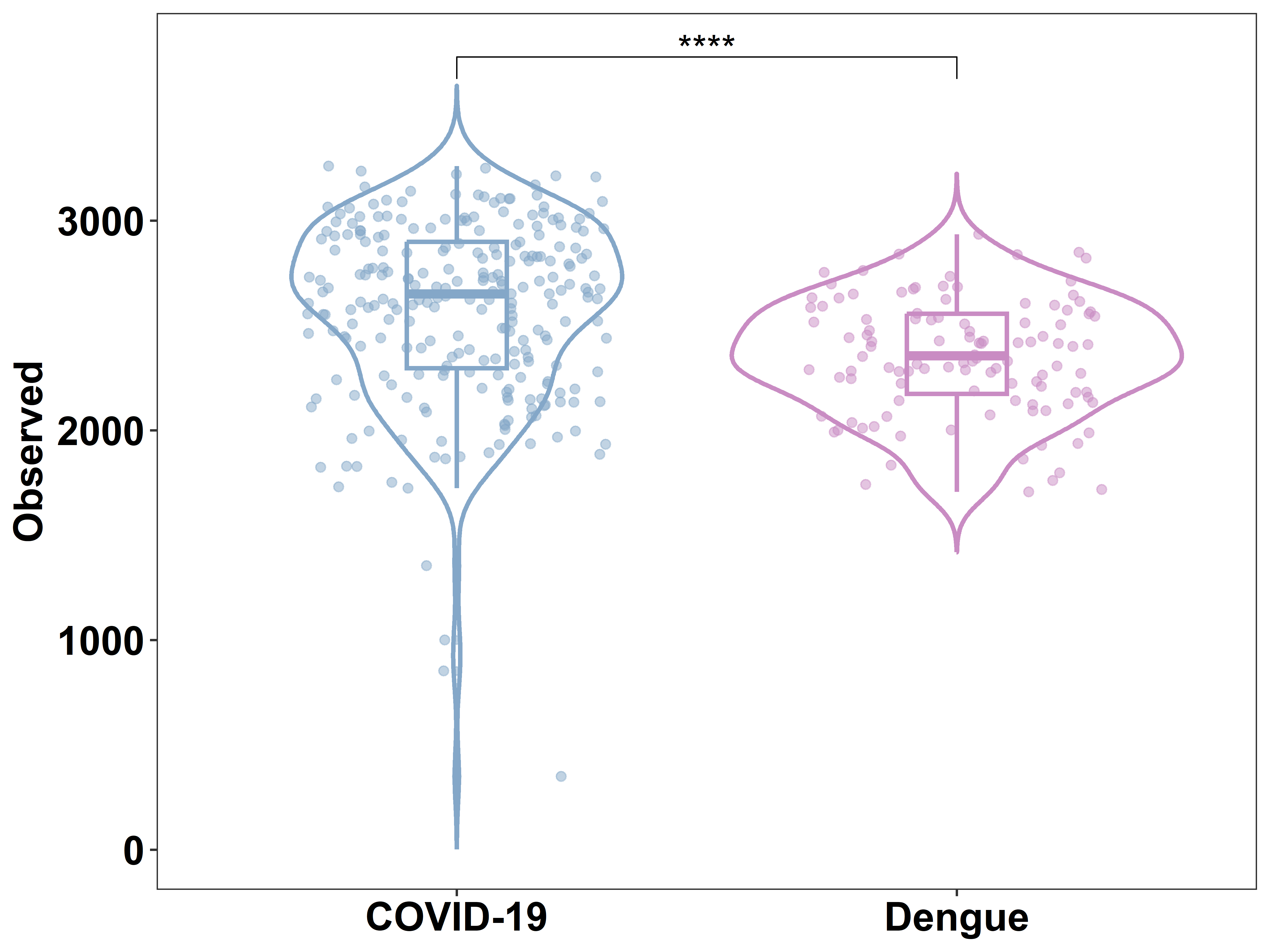


iii.


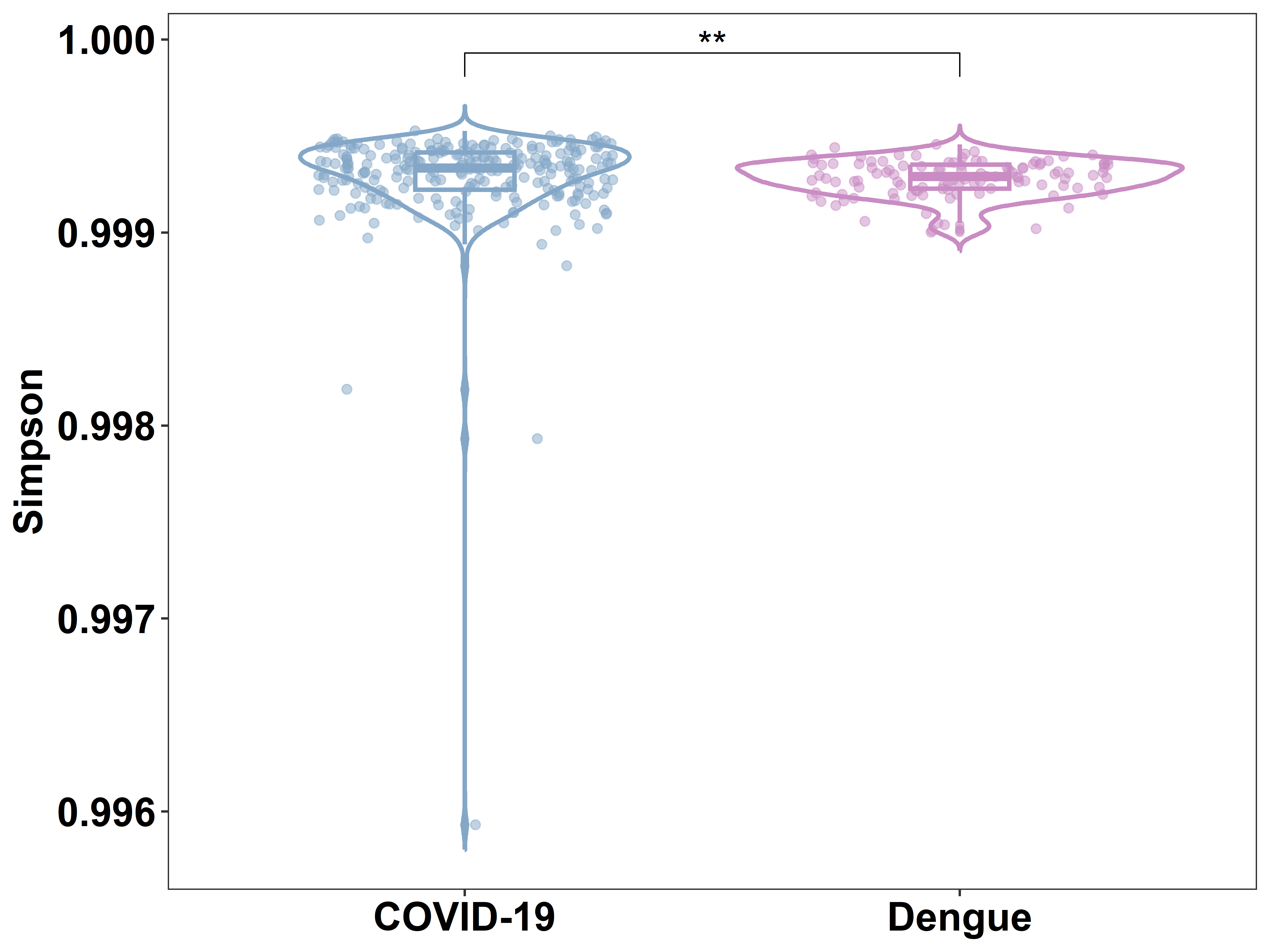


**Figure:** Alpha diversity index for (a) ARGs and (b) TAMs in COVID-19 (blue) and dengue (pink). (i) Chao1 (ii) Observed (iii) Simpson indices.
