# Supplementary material for: Non-canonical Metatranscriptomic analysis of COVID-19 and Dengue reveals an expanded microbial and AMR landscape in COVID-19 mortality patients
Source: PLoS Pathog. 2025 Nov 19;21(11):e1013703. doi: 10.1371/journal.ppat.1013703 (PMC12629440; doi:10.1371/journal.ppat.1013703)
Supplement: S3 File — (DOCX) [file ppat.1013703.s003.docx]

**Non-canonical Metatranscriptomic analysis of COVID-19 and Dengue reveals an expanded microbial and AMR landscape in COVID-19 mortality patients**

Aanchal Yadav^1,3,6^, Raiyan Ali^1,6^, Priti Devi^1,3^, Pallawi Kumari^1,4^, Jyoti Soni^1,3^, Garima^1,3^, Bansidhar Tarai^5^, Sandeep Budhiraja^5^, Uzma Shamim^1,2,*^ , Rajesh Pandey^1,3,7,*^

^1^Division of Immunology and Infectious Disease Biology, INtegrative GENomics of HOst-PathogEn (INGEN-HOPE) laboratory, CSIR-Institute of Genomics and Integrative Biology (CSIR-IGIB), Mall Road, Delhi-110007, India.

^2^Ashoka University, Sonipat, Haryana-131029, India

^3^Academy of Scientific and Innovative Research (AcSIR), Ghaziabad-201002, India.

^4^Indraprastha Institute of Information Technology (IIIT), New Delhi-110020, India

^5^Max Super Speciality Hospital (A Unit of Devki Devi Foundation), Max Healthcare, Delhi 110017, India.

^6^Equal contribution

^*^Co-corresponding authors

^7^Lead contact

Contact Details:

**Rajesh Pandey, PhD**

Principal Scientist,

INtegrative GENomics of HOst-PathogEn (INGEN-HOPE) laboratory,

CSIR-Institute of Genomics and Integrative Biology (CSIR-IGIB),

North Campus, Near Jubilee Hall, Mall Road, Delhi-110007, India.

Contact: [rajeshp@igib.in](mailto:rajeshp@igib.in); [rajesh.p@igib.res.in](mailto:rajesh.p@igib.res.in); Tel.: 011-27002200 (Ext. 254)

**Running title:** Resistome and Microbiome Dynamics in COVID-19 and Dengue

**Supplementary File S3: Comparative analysis for concordance of ARGs and microbial abundance between two independent dengue cohorts for validation of study findings.**

To ensure robustness and reproducibility of our finding, we analysed a separate in-house study involving an independent cohort of 58 dengue patients, of whom 26 had severe dengue and 32 had non-severe dengue, categorized according to WHO guidelines, for the presence of ARGs and microbial community composition. Same methodological framework was used for this dataset as well.

Specifically, we compared antimicrobial resistance genes (ARGs) between the discovery (n=112) and validation (n=58) cohorts and identified 300 total ARGs, with 208 shared across both the cohorts **(Figure 1a).** To further enhance the stringency and reliability of the comparative analysis, we applied uniform abundance and coverage thresholds to both cohorts: only ARGs with ≥30% gene coverage present in ≥10% of samples, and TAMs with ≥0.1% relative abundance in at least 50% of samples, were considered. The results of analysis showed strong overlap between cohorts, with high correlation for ARGs abundance (R² = 0.93, p = 0.0067) **(Figure 1b)** and microbial composition (R² = 0.77, p < 2.2 × 10⁻¹⁶) **(Figure 1c)**. This analysis further validated the results of current study, confirming that ARGs and microbial signatures among the cohorts are reproducible and biologically consistent, thereby strengthening the validity of resistome landscape described in this study.


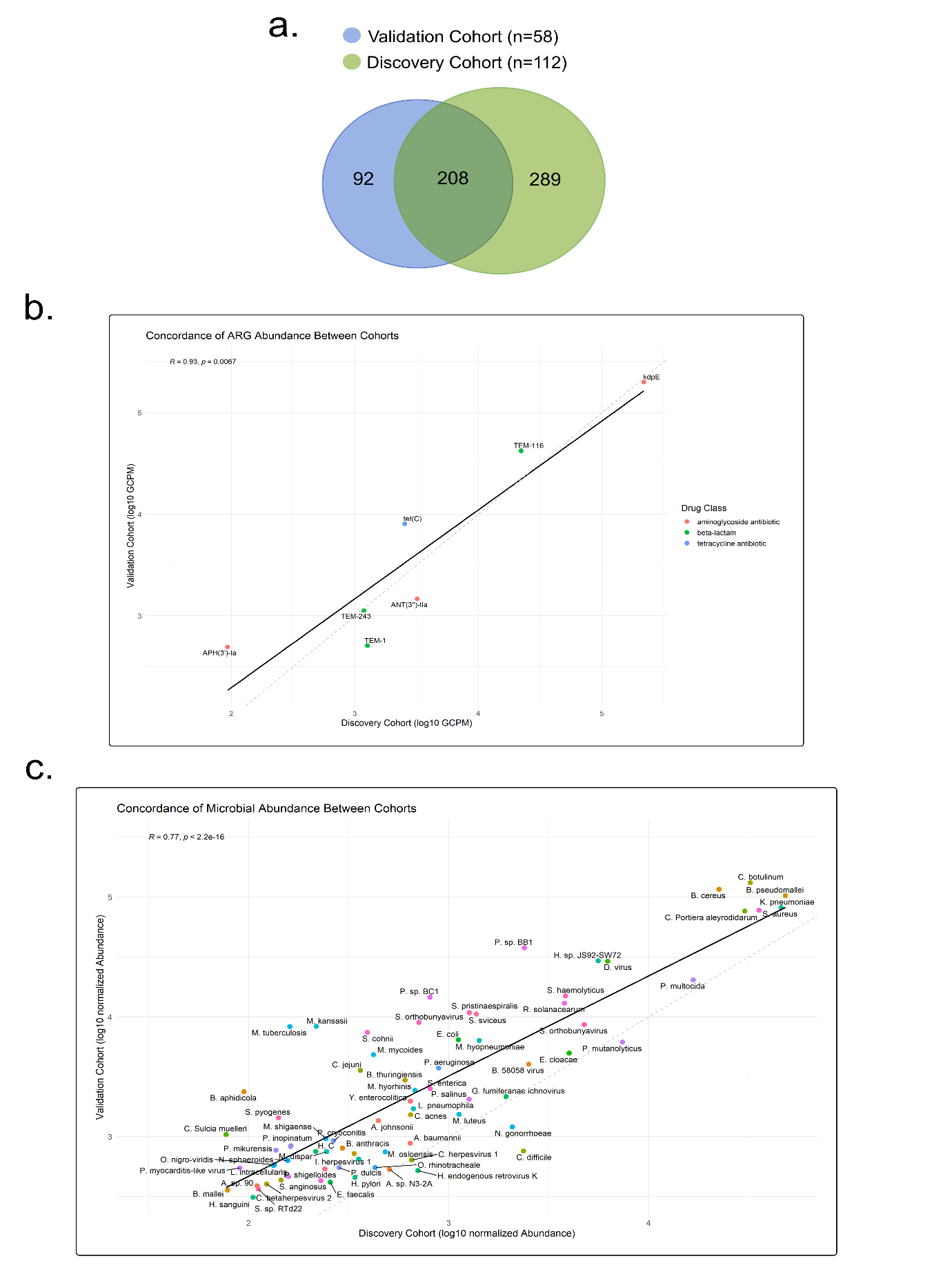


**Figure:** (a) Venn diagram showing overlapping ARGs between validation cohort and discovery cohort. Concordance plots showing (a) concordance of ARGs abundance and, (c) concordance of microbial abundance between two independent cohorts.
