# Supplementary material for: Non-canonical Metatranscriptomic analysis of COVID-19 and Dengue reveals an expanded microbial and AMR landscape in COVID-19 mortality patients
Source: PLoS Pathog. 2025 Nov 19;21(11):e1013703. doi: 10.1371/journal.ppat.1013703 (PMC12629440; doi:10.1371/journal.ppat.1013703)
Supplement: S4 File — (DOCX) [file ppat.1013703.s004.docx]

**Non-canonical Metatranscriptomic analysis of COVID-19 and Dengue reveals an expanded microbial and AMR landscape in COVID-19 mortality patients**

Aanchal Yadav^1,3,6^, Raiyan Ali^1,6^, Priti Devi^1,3^, Pallawi Kumari^1,4^, Jyoti Soni^1,3^, Garima^1,3^, Bansidhar Tarai^5^, Sandeep Budhiraja^5^, Uzma Shamim^1,2,*^ , Rajesh Pandey^1,3,7,*^

^1^Division of Immunology and Infectious Disease Biology, INtegrative GENomics of HOst-PathogEn (INGEN-HOPE) laboratory, CSIR-Institute of Genomics and Integrative Biology (CSIR-IGIB), Mall Road, Delhi-110007, India.

^2^Ashoka University, Sonipat, Haryana-131029, India

^3^Academy of Scientific and Innovative Research (AcSIR), Ghaziabad-201002, India.

^4^Indraprastha Institute of Information Technology (IIIT), New Delhi-110020, India

^5^Max Super Speciality Hospital (A Unit of Devki Devi Foundation), Max Healthcare, Delhi 110017, India.

^6^Equal contribution

^*^Co-corresponding authors

^7^Lead contact

Contact Details:

**Rajesh Pandey, PhD**

Principal Scientist,

INtegrative GENomics of HOst-PathogEn (INGEN-HOPE) laboratory,

CSIR-Institute of Genomics and Integrative Biology (CSIR-IGIB),

North Campus, Near Jubilee Hall, Mall Road, Delhi-110007, India.

Contact: [rajeshp@igib.in](mailto:rajeshp@igib.in); [rajesh.p@igib.res.in](mailto:rajesh.p@igib.res.in); Tel.: 011-27002200 (Ext. 254)

**Running title:** Resistome and Microbiome Dynamics in COVID-19 and Dengue

**Supplementary File S4: Genome Coverage of Dengue Virus and SARS-CoV-2 Across Patient Samples**

Mapping of sequencing reads to the Dengue virus and SARS-CoV-2 reference genomes was performed to verify the presence of primary pathogens. This quality control step confirms successful capture of host-pathogen dynamics in the meta-transcriptomic data and supports downstream analyses based on microbial transcriptional activity.

Dengue viral reads are found to be consistently detected across all the samples with genome coverage ranging from 17% to 100% with around 72 of these samples exhibited the viral read coverage of >90%.

For COVID-19 patient samples, significant reads of SARS-CoV-2 reads are detected in samples with 163 samples having >50% viral read coverage. Majority of samples with viral coverage above 50% gives the confidence and confirmation over the patient samples.

Both Dengue virus and SARS-CoV-2, being positive-sense single-stranded RNA viruses, showed substantial alignment to their reference genomes, confirming effective capture of viral sequences in the meta-transcriptomic data throughout the respective patient samples.

a.


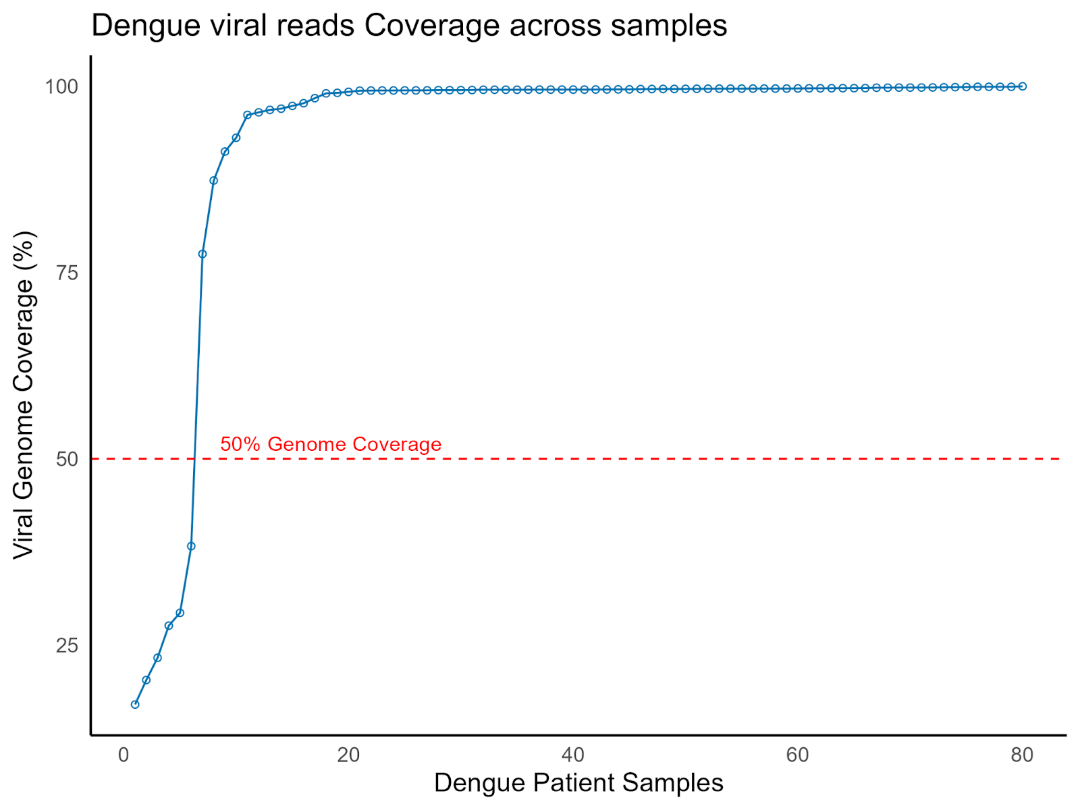


b.


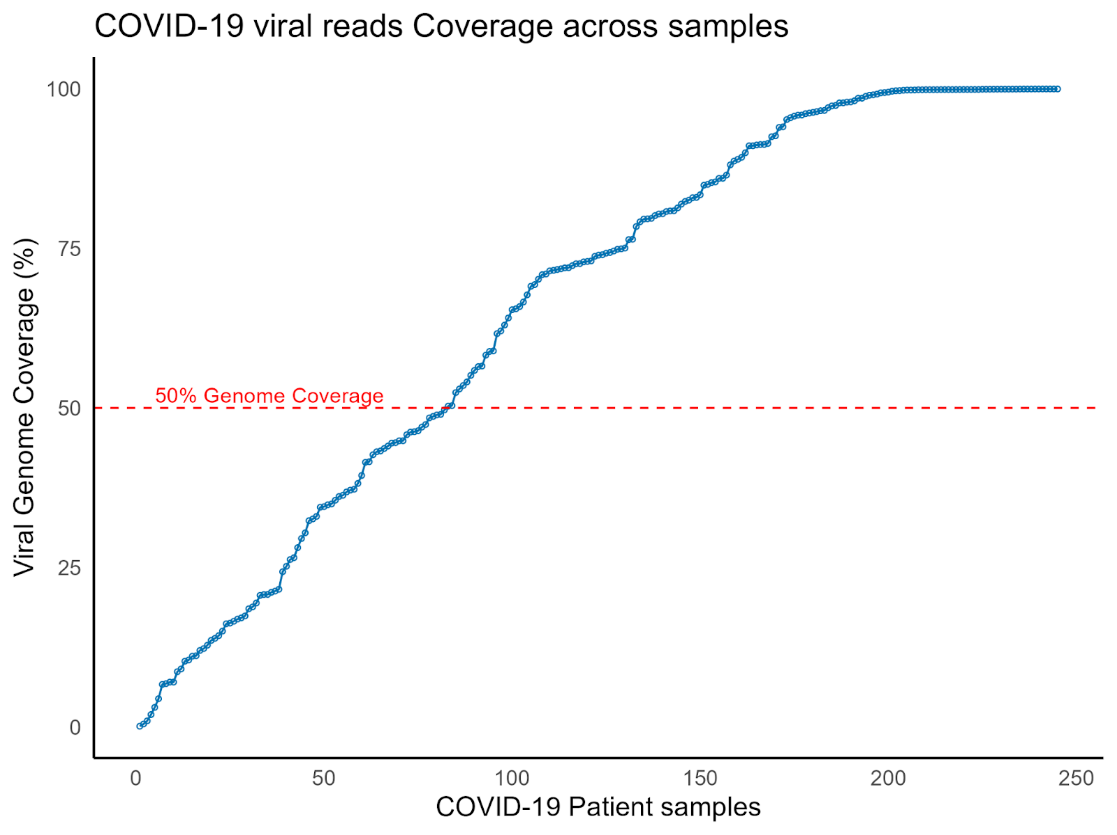


**Figure.** Viral RNA genome coverage across patient samples: (a) Dengue virus in dengue-positive patients, (b) SARS-CoV-2 in COVID-19 patients.
