# Supplementary material for: Non-canonical Metatranscriptomic analysis of COVID-19 and Dengue reveals an expanded microbial and AMR landscape in COVID-19 mortality patients
Source: PLoS Pathog. 2025 Nov 19;21(11):e1013703. doi: 10.1371/journal.ppat.1013703 (PMC12629440; doi:10.1371/journal.ppat.1013703)
Supplement: S5 File — (DOCX) [file ppat.1013703.s005.docx]

**Non-canonical Metatranscriptomic analysis of COVID-19 and Dengue reveals an expanded microbial and AMR landscape in COVID-19 mortality patients**

Aanchal Yadav^1,3,6^, Raiyan Ali^1,6^, Priti Devi^1,3^, Pallawi Kumari^1,4^, Jyoti Soni^1,3^, Garima^1,3^, Bansidhar Tarai^5^, Sandeep Budhiraja^5^, Uzma Shamim^1,2,*^ , Rajesh Pandey^1,3,7,*^

^1^Division of Immunology and Infectious Disease Biology, INtegrative GENomics of HOst-PathogEn (INGEN-HOPE) laboratory, CSIR-Institute of Genomics and Integrative Biology (CSIR-IGIB), Mall Road, Delhi-110007, India.

^2^Ashoka University, Sonipat, Haryana-131029, India

^3^Academy of Scientific and Innovative Research (AcSIR), Ghaziabad-201002, India.

^4^Indraprastha Institute of Information Technology (IIIT), New Delhi-110020, India

^5^Max Super Speciality Hospital (A Unit of Devki Devi Foundation), Max Healthcare, Delhi 110017, India.

^6^Equal contribution

^*^Co-corresponding authors

^7^Lead contact

Contact Details:

**Rajesh Pandey, PhD**

Principal Scientist,

INtegrative GENomics of HOst-PathogEn (INGEN-HOPE) laboratory,

CSIR-Institute of Genomics and Integrative Biology (CSIR-IGIB),

North Campus, Near Jubilee Hall, Mall Road, Delhi-110007, India.

Contact: [rajeshp@igib.in](mailto:rajeshp@igib.in); [rajesh.p@igib.res.in](mailto:rajesh.p@igib.res.in); Tel.: 011-27002200 (Ext. 254)

**Running title:** Resistome and Microbiome Dynamics in COVID-19 and Dengue

**Supplementary File S5: Evaluation of Bracken-Derived TAMs Relative Abundance Estimates Using Multiple Calculation Methods**

To assess the robustness of Bracken-based microbial abundance estimates, we reanalyzed the data using three different methods: (a) direct use of raw and normalized read counts from Bracken, (b) manual calculation of relative abundance from raw reads, and (c) relative abundance based on normalized counts. Across all methods, the microbial distribution patterns and top taxa remained consistent, highlighting the reliability and efficiency of Bracken for relative abundance estimation in metatranscriptomic data.


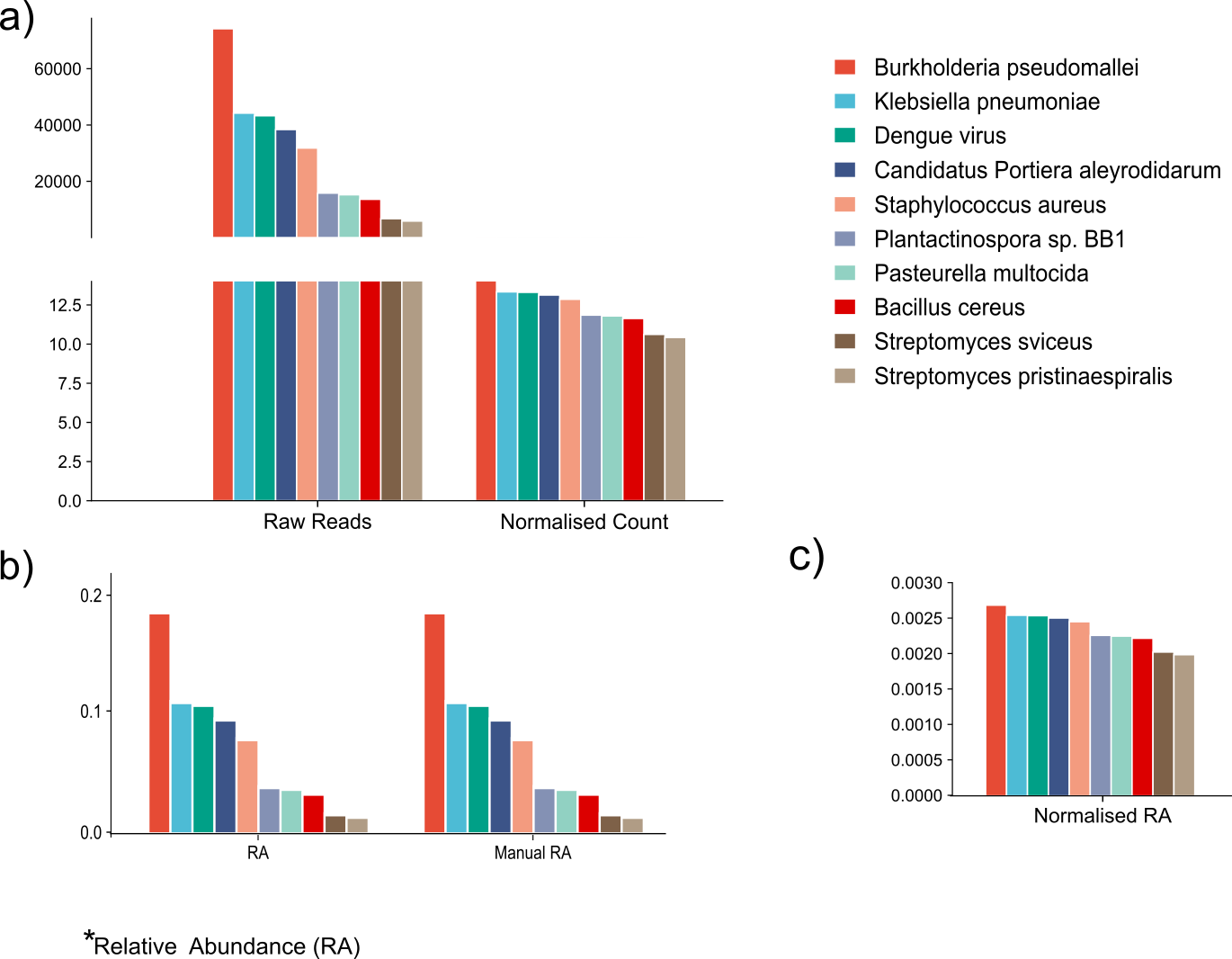


**Figure.** Comparison of microbial relative abundance profiles derived using different methods: (a) Bracken raw and normalized read counts, (b) manually computed relative abundance from raw reads, and (c) normalized read-based calculations. The consistent profiles across methods demonstrate the robustness of abundance estimation.
