# Supplementary material for: Non-canonical Metatranscriptomic analysis of COVID-19 and Dengue reveals an expanded microbial and AMR landscape in COVID-19 mortality patients
Source: PLoS Pathog. 2025 Nov 19;21(11):e1013703. doi: 10.1371/journal.ppat.1013703 (PMC12629440; doi:10.1371/journal.ppat.1013703)
Supplement: S6 File — (DOCX) [file ppat.1013703.s006.docx]

**Non-canonical Metatranscriptomic analysis of COVID-19 and Dengue reveals an expanded microbial and AMR landscape in COVID-19 mortality patients**

Aanchal Yadav^1,3,6^, Raiyan Ali^1,6^, Priti Devi^1,3^, Pallawi Kumari^1,4^, Jyoti Soni^1,3^, Garima^1,3^, Bansidhar Tarai^5^, Sandeep Budhiraja^5^, Uzma Shamim^1,2,*^ , Rajesh Pandey^1,3,7,*^

^1^Division of Immunology and Infectious Disease Biology, INtegrative GENomics of HOst-PathogEn (INGEN-HOPE) laboratory, CSIR-Institute of Genomics and Integrative Biology (CSIR-IGIB), Mall Road, Delhi-110007, India.

^2^Ashoka University, Sonipat, Haryana-131029, India

^3^Academy of Scientific and Innovative Research (AcSIR), Ghaziabad-201002, India.

^4^Indraprastha Institute of Information Technology (IIIT), New Delhi-110020, India

^5^Max Super Speciality Hospital (A Unit of Devki Devi Foundation), Max Healthcare, Delhi 110017, India.

^6^Equal contribution

^*^Co-corresponding authors

^7^Lead contact

Contact Details:

**Rajesh Pandey, PhD**

Principal Scientist,

INtegrative GENomics of HOst-PathogEn (INGEN-HOPE) laboratory,

CSIR-Institute of Genomics and Integrative Biology (CSIR-IGIB),

North Campus, Near Jubilee Hall, Mall Road, Delhi-110007, India.

Contact: [rajeshp@igib.in](mailto:rajeshp@igib.in); [rajesh.p@igib.res.in](mailto:rajesh.p@igib.res.in); Tel.: 011-27002200 (Ext. 254)

**Running title:** Resistome and Microbiome Dynamics in COVID-19 and Dengue

**Supplementary File S6: Cumulative Contribution of TAMs with >0.1% Relative Abundance to the Total Abundance Within the Corresponding Phyla.**

The threshold of >0.1% relative abundance for defining transcriptionally active microbes (TAMs) was selected to capture species making a meaningful contribution to their respective phyla, even if their individual abundances were modest. To justify this cutoff, we quantified the cumulative contribution of all species exceeding the 0.1% threshold within each phylum. Then we checked the percentage of cumulative species (belonging to each phylum) with respect total species abundance within each phylum. The count percentage indicates the contribution of abundant species in their respective phylum.

This analysis demonstrated that, although the threshold may seem low in absolute terms, the included species collectively represent a substantial—and often dominant—proportion of the total phylum-level abundance across samples.

Table (a): **Percentage of species with exceeding 0.1% relative abundance present in 50% of sample to total phylum-level abundance in dengue samples**

| **Dengue Abundant Phyla** | **Species with >0.1% RA in 50% of sample** | **Total species in phyla** | **Counts Percentage** |
| --- | --- | --- | --- |
| *Actinobacteria* | 17 | 608 | 2.8 |
| *Artverviricota* | 1 | 39 | 2.6 |
| *Bacteroidetes* | 3 | 285 | 1.1 |
| *Cyanobacteria* | 4 | 122 | 3.3 |
| *Duplornaviricota* | 1 | 25 | 4.0 |
| *Euryarchaeota* | 3 | 170 | 1.8 |
| *Firmicutes* | 16 | 625 | 2.6 |
| *Kitrinoviricota* | 2 | 109 | 1.8 |
| *Negarnaviricota* | 2 | 128 | 1.6 |
| *Nucleocytoviricota* | 1 | 105 | 1.0 |
| *Peploviricota* | 5 | 83 | 6.0 |
| *Pisuviricota* | 1 | 155 | 0.6 |
| *Planctomycetes* | 1 | 17 | 5.9 |
| *Proteobacteria* | 48 | 1795 | 2.7 |
| *Spirochaetes* | 1 | 48 | 2.1 |
| *Tenericutes* | 10 | 95 | 10.5 |
| NA | 5 | 367 | 1.4 |

Table (b): **Percentage of species with exceeding 0.1% relative abundance present in 50% of sample to total phylum-level abundance in COVID-19 samples**

| **COVID-19 Abundant Phyla** | **Species with >0.1% RA in 50% of sample** | **Total species in phyla** | **Counts Percentage** |
| --- | --- | --- | --- |
| *Actinobacteria* | 11 | 610 | 1.8 |
| *Bacteroidetes* | 7 | 285 | 2.5 |
| *Firmicutes* | 24 | 628 | 3.8 |
| *Fusobacteria* | 3 | 17 | 17.6 |
| *Negarnaviricota* | 2 | 215 | 0.9 |
| *Proteobacteria* | 30 | 1806 | 1.7 |
| *Tenericutes* | 3 | 95 | 3.2 |
