# Supplementary material for: Non-canonical Metatranscriptomic analysis of COVID-19 and Dengue reveals an expanded microbial and AMR landscape in COVID-19 mortality patients
Source: PLoS Pathog. 2025 Nov 19;21(11):e1013703. doi: 10.1371/journal.ppat.1013703 (PMC12629440; doi:10.1371/journal.ppat.1013703)
Supplement: S7 File — (DOCX) [file ppat.1013703.s007.docx]

**Non-canonical Metatranscriptomic analysis of COVID-19 and Dengue reveals an expanded microbial and AMR landscape in COVID-19 mortality patients**

Aanchal Yadav^1,3,6^, Raiyan Ali^1,6^, Priti Devi^1,3^, Pallawi Kumari^1,4^, Jyoti Soni^1,3^, Garima^1,3^, Bansidhar Tarai^5^, Sandeep Budhiraja^5^, Uzma Shamim^1,2,*^ , Rajesh Pandey^1,3,7,*^

^1^Division of Immunology and Infectious Disease Biology, INtegrative GENomics of HOst-PathogEn (INGEN-HOPE) laboratory, CSIR-Institute of Genomics and Integrative Biology (CSIR-IGIB), Mall Road, Delhi-110007, India.

^2^Ashoka University, Sonipat, Haryana-131029, India

^3^Academy of Scientific and Innovative Research (AcSIR), Ghaziabad-201002, India.

^4^Indraprastha Institute of Information Technology (IIIT), New Delhi-110020, India

^5^Max Super Speciality Hospital (A Unit of Devki Devi Foundation), Max Healthcare, Delhi 110017, India.

^6^Equal contribution

^*^Co-corresponding authors

^7^Lead contact

Contact Details:

**Rajesh Pandey, PhD**

Principal Scientist,

INtegrative GENomics of HOst-PathogEn (INGEN-HOPE) laboratory,

CSIR-Institute of Genomics and Integrative Biology (CSIR-IGIB),

North Campus, Near Jubilee Hall, Mall Road, Delhi-110007, India.

Contact: [rajeshp@igib.in](mailto:rajeshp@igib.in); [rajesh.p@igib.res.in](mailto:rajesh.p@igib.res.in); Tel.: 011-27002200 (Ext. 254)

**Running title:** Resistome and Microbiome Dynamics in COVID-19 and Dengue

**Supplementary File S7: Distribution of Microbial ARG Coverage and Multi-/Single-Site ARGs Within the 30–50% Threshold Range.**

Antibiotic resistance genes (ARGs) exhibiting >30% coverage and present in at least 10% of the samples are graphically represented. In dengue samples, the distribution of ARGs appears relatively continuous across the 30–50% coverage threshold. In contrast, COVID-19 samples show a marked decline in the number of ARGs as coverage increases from 30% to 50%. To investigate this further, ARGs within the 30–50% coverage range were examined to determine whether their coverage was restricted to single genomic regions or spanned multiple, non-contiguous regions (multi-site coverage). Within this coverage range, 92 ARGs in COVID-19 and 14 in dengue were uniquely identified.

These ARGs were further analyzed for their RNA-seq read mapping patterns. A substantial proportion exhibited read coverage across multiple, non-adjacent regions of the gene loci, including the start, middle, and end' regions. This pattern supports the conclusion that the observed partial coverage is not due to random or non-specific mapping artifacts, but rather reflects authentic transcriptional activity.

To assess the spatial distribution of read coverage, ARGs were plotted to visualize their site-level occurrence in both disease conditions. Some ARGs are selected to illustrate both single-site and multi-site coverage patterns as an example. This spatial coverage analysis reinforces the confidence that reads are mapping to significant portions of the gene sequences, supporting their inclusion in subsequent analyses.

**
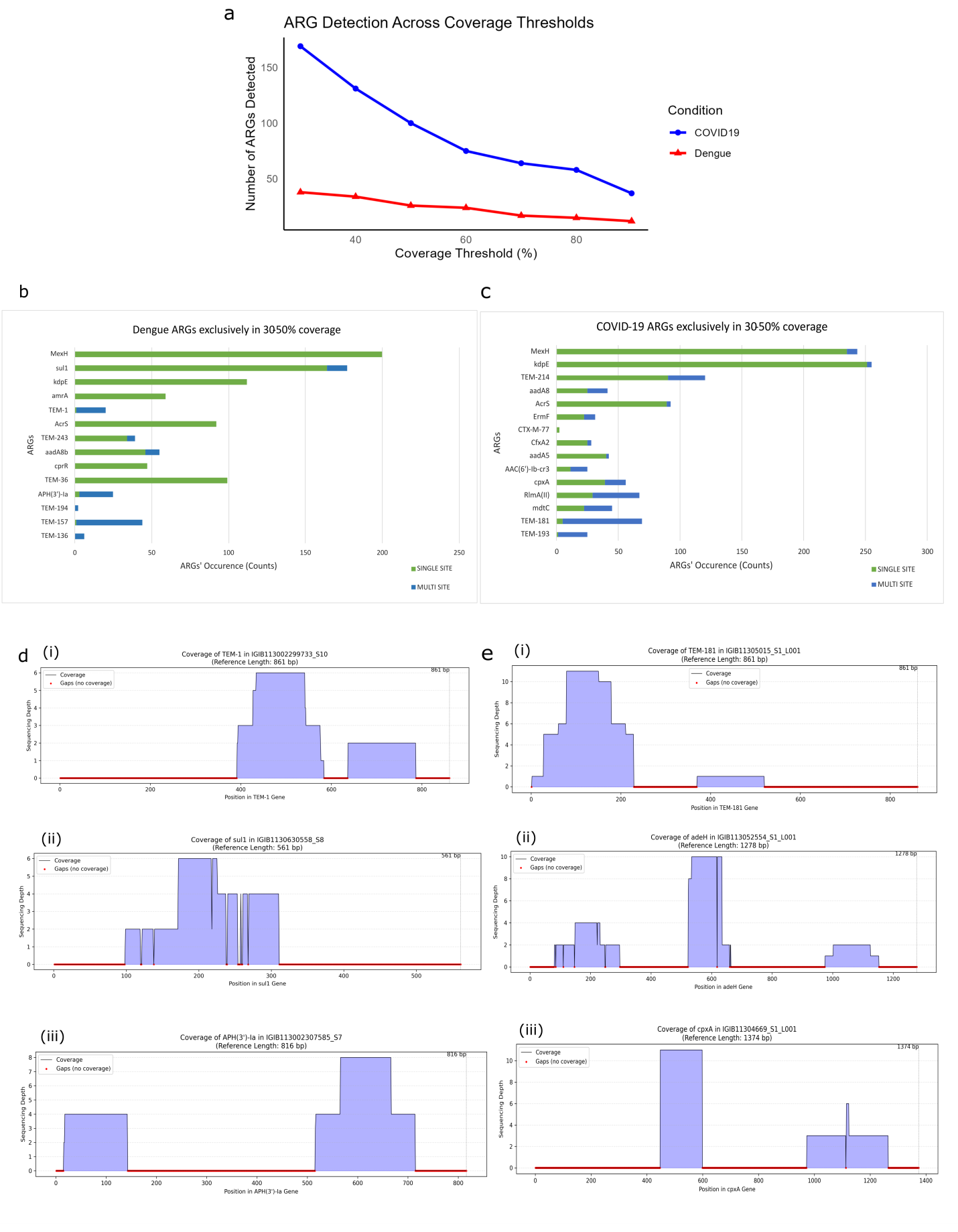
**

**Figure:** (a) Number of ARGs detected across increasing coverage thresholds (30% to 90%). (b) Site-wise distribution of ARGs with 30–50% gene coverage in (b) dengue samples, (c) COVID-19 samples. (d) Read coverage across multiple sites of selected ARGs in dengue: (i) *TEM-1*, (ii) *Sul1*, and (iii) *APH(3’)-Ia*. (e) Read coverage across selected ARGs in COVID-19: (i) *TEM-181*, (ii) *adeH*, and (iii) *cpxA*.
