# Supplementary material for: Non-canonical Metatranscriptomic analysis of COVID-19 and Dengue reveals an expanded microbial and AMR landscape in COVID-19 mortality patients
Source: PLoS Pathog. 2025 Nov 19;21(11):e1013703. doi: 10.1371/journal.ppat.1013703 (PMC12629440; doi:10.1371/journal.ppat.1013703)
Supplement: Attachment — Submitted filename: Response to Reviewers.docx [file ppat.1013703.s015.docx]

**Response to Reviewers**

**Reviewer #1 -**

**Part I - Summary**
Reviewer #1: This is a well written manuscript that tackles and important and, mostly, unanswered question in AMR- how does the viral species (ssRNA based in this case) interact with the bacterial resistome. The study explored the burden of resistance between SARS-CoV-2 and Dengue patients and the microbiome, focusing on the transcriptionally active microbes and antimicrobial resistance genes.

The study uses large patient cohorts (COVID19- 251 patients, and Dengue 112 patients) which strengthens some of the statistical power and clinical relevance of the findings. However, there are some key limitations within the study that indicate some overstated claims without substantial evidence to support these with the data. There are some methodological limitations and lack of clarity with use of controls and possible biases in sampling.
However, the data sets are of interest to the AMR community and with major revisions the content would improve.

In terms of presentations, there is a lack of use of citations throughout to support methodologies and claims made. Adding suitable citations would scientifically improve the robustness of the manuscript. There are changes in font throughout the text, and use of abbreviations which have not been defined throughout. Addressing these would enhance readability.

We thank the Reviewer for the balanced feedback combining study summarization, constructive feedback and suggestions to improve further. We are pleased that the significance of our study—exploring the interaction between ssRNA viral infections (SARS-CoV-2 and Dengue) and the transcribed bacterial resistome—was recognized, along with the strength provided by the large patient cohorts. In response to the concerns raised, we have revised the manuscript to address methodological clarity, including detailed descriptions of the steps taken to reduce bias. We have moderated claims to more accurately reflect the data, distinguishing observed associations from potential causation. Appropriate citations have been added to support our methodology and key statements, enhancing scientific rigor. Furthermore, we have corrected all formatting inconsistencies and ensured that all abbreviations are defined at their first mention to improve readability. We believe these revisions significantly improve the clarity and robustness of the manuscript.

**Part II – Major Issues: Key Experiments Required for Acceptance**
Reviewer #1:

1. The authors refer to a "non-canonical metatranscriptomics" approach to describe the methods used; however this term is undefined and not supported by a clear method when compared to standard metagenomic workflows and pipelines. Explaining this methodology, and its differences to standard metagenomics, would be helpful. There is no information about the specific bioinformatics software tools, benchmarking or validation of this approach; therefore it is not easy to assess the reproducibility and validity of this approach.

We thank the reviewer for this valuable observation and for prompting us to further improve the clarity and transparency of our methods. We agree that the term **“non-canonical metatranscriptomics”** was not clearly defined in the original manuscript. In the revised version, we have now explicitly clarified this term and the rationale behind our approach:

- **Term clarification**: By “non-canonical metatranscriptomics,” we refer to the repurposing of host-transcriptome total RNA-seq data—originally generated for host transcriptomics studies— to identify and quantify transcriptionally active microbes (TAMs) in this study. Unlike traditional metatranscriptomic approaches, which involve RNA extraction from isolated microbial communities and targeted microbial enrichment, our method starts with total RNA extracted from clinical human samples and applies computational filtering to remove host-aligned reads post-sequencing. The reason we believe it is a better way to capture the host and microbial interactome is because we are doing no preferential enrichment or depletion for either human host or microbial communities, but capturing the natural dynamics.
- **Comparison with standard methods**: This strategy differs fundamentally from standard metagenomics, which relies on DNA sequencing and cannot distinguish between active and dormant microbes, and from conventional metatranscriptomics, which typically enriches microbial RNA prior to sequencing. Our approach avoids such enrichment steps, instead relying on native total RNA, and removes host reads computationally to analyze microbial expression in its in-vivo context.
- **Biological relevance**: We believe this approach provides a more unbiased and representative snapshot of microbial activity. Enrichment protocols can introduce experimental bias, disproportionately affecting microbial transcript abundance and potentially skewing results—particularly in low-biomass, host-dominated samples like blood or serum. In contrast, our method preserves the native biological state of the sample. We observed disease-specific differences in leftover microbial reads—COVID-19 samples showed ~58% human reads, while Dengue samples had ~92%—indicating that these signals reflect true biological variation rather than technical noise. This strengthens our strategy in the value of analyzing these “leftover/unmapped” reads to uncover disease-informative transcriptionally active microbial signatures.
- **Reproducibility and transparency**: To ensure reproducibility, we have now provided a more detailed description of our computational pipeline in the revised Methods section. Additionally, a complete experimental and analytical protocol has been previously published as a STAR Protocol (Yadav et al., STAR Protocols, 2023; DOI: [10.1016/j.xpro.2024.103071](https://doi.org/10.1016/j.xpro.2024.103071)), in addition to the original research articles using those methodologies, which we have now referenced and incorporated into the revised manuscript.

All the above points have now been incorporated in the revised manuscript as follows:

In the Introduction section, we have added:

*“In this study, we employed a previously lab used “non-canonical meta-transcriptomics” approach, which we define as the repurposing of host total RNA-seq data—originally generated for the host transcriptomics—to identify and quantify transcriptionally active microbes (TAMs). Unlike conventional metagenomics or metatranscriptomics, which involve microbial DNA/RNA enrichment, our method starts from native clinical samples based on disease manifestation site (e.g., blood or nasopharyngeal swabs for Dengue and COVID-19) and removes host-aligned reads computationally to analyze microbial expression. This strategy enables unbiased profiling of active microbial communities without introducing potential experimental biases from the enrichment protocols, which can distort transcript abundance in low-biomass, host-dominated samples.”*

In the Result 1, we have added:

*“We observed clear disease-specific differences in the microbial signal, with ~58% host reads in the COVID-19 samples and ~92% in the dengue samples, supporting the biological relevance of the leftover microbial reads analyzed in this study.”*

In the Method section, we have added:

*“A detailed description of the experimental and analytical pipeline is available in our published STAR Protocol (*[*https://doi.org/10.1016/j.xpro.2024.103071*](https://doi.org/10.1016/j.xpro.2024.103071)*), ensuring full reproducibility of the methods used.”*

2. There is a lack of detail in the manuscript with regards to variables that are known to already influence the microbiome, for example associated with diet, antibiotic use and clinical information.

We thank the reviewer for highlighting the importance of host-related variables that may influence the microbiome, such as diet, antibiotic use, and clinical information.

- **Diet:**
  Due to the emergency clinical setting during the COVID-19 pandemic, we were unable to collect detailed dietary information from the participants. We acknowledge that it would have been perfect to gather dietary information as well and thus included that in the potential limitations in the revised submission, which now read as:

“*Another potential limitation is the absence of dietary information, which could be a confounding factor; however, current evidence indicates that diet has minimal direct influence on the blood and nasal microbiomes*.”

Additionally, we have incorporated the following text in the discussion section to address the diet related comment:

“*It is important to point out that diet is a major modulator of the gut microbiome, however, its direct impact on the blood and nasal microbiomes (based on the studies diseases) is minimal and largely indirect. Unlike the gut, there is limited evidence linking dietary patterns to microbial composition in these sites (*[*https://doi.org/10.1016/j.nmni.2021.100845*](https://doi.org/10.1016/j.nmni.2021.100845)*). Most dietary influences on host health are mediated through gut-derived metabolites that circulate systemically, rather than through direct microbial colonization of blood or nasal tissues (*[*https://doi.org/10.1038/nm.3444*](https://doi.org/10.1038/nm.3444)*,* [*https://doi.org/10.1126/scitranslmed.3009759*](https://doi.org/10.1126/scitranslmed.3009759)*). Since our study focuses on microbiomes at the primary sites of infection—nasopharyngeal swabs for COVID-19 and blood for Dengue—dietary effects are unlikely to significantly confound our findings, though a minor influence cannot be entirely excluded.*”

- **Antibiotic Use:**

We acknowledge the importance of antibiotic exposure as a potential confounding factor in AMR analyses. To augment clarification vis-à-vis our study, all samples were collected on the first day of hospital reporting—prior to admission and before initiation of any in-hospital treatment. Therefore, antibiotic administration within the hospital setting did not influence the samples used for meta-transcriptomic analysis. However, since we did not prospectively capture patients’ pre-hospital antibiotic use history (i.e., antibiotics taken before arriving at the hospital), this remains a limitation for a subset of cases. Given that our AMR findings are based on first-contact, treatment-naïve samples, we believe the immediate impact of antibiotics on the observed resistome is minimal.

Initially, we excluded this variable due to incomplete data across both the cohorts. While partial antibiotic usage data were available for COVID-19 patients, these data were not collected for dengue patients.

To address the reviewer’s query, we have now added the available antibiotic information for COVID-19 patients as a **“Supplementary Table S1”** in the revised manuscript, and is placed here for reference.

**Supplementary Table S1: Antibiotic Data for COVID-19 Patients (n=145)**

| **S.No.** | **Antibiotics** | **COVID-19 Disease Severity (%)** | | | | **p-values** |
| --- | --- | --- | --- | --- | --- | --- |
|  |  | **Mild (n=128)** | **Moderate (n=33)** | **Severe (n=24)** | **Mortality(n=60)** |  |
| **1** | **Antiviral** | 47 (36.7) | 12 (36.4) | 9 (36) | 3 (5) | 0.000059 |
| **2** | **Anti-inflammatory** | 21 (16.4) | 2 (6.1) | 2 (8) | 5 (8.3) | 0.218311 |
| **3** | **Anticoagulant** | 16 (12.5) | 6 (18.2) | 0 | 0 | 0.396821 |
| **4** | **Antibacterial** | 53 (41.4) | 9 (27.3) | 9 (36) | 4 (6.7) | 0.000026 |
| **4A** | **Beta-lactams** | 5(3.9) | 3 (9.1) | 3(12.5) | 0 | 0.184581 |
| **4B** | **Tetracycline** | 18(14.1) | 2(6.1) | 2(8.3) | 0 | 0.379726 |
| **4C** | **Macrolides** | 29(22.7) | 6(18.2) | 5(20.8) | 4 (6.7) | 0.064477 |
| **4D** | **Nitro imidazole** | 0 | 1 (3) | 0 | 0 |  |
| **4E** | **Oxazolidinone** | 0 | 0 | 1(4.2) | 0 |  |

Additionally, we have now incorporated antibiotic-related information throughout the revised manuscript, including in the *Results*, *Discussion*, and *Limitations* sections, as follows:

**Result 1:** “*In our study, all samples from both the COVID-19 and dengue patient groups were collected on the first day of hospital reporting, prior to admission and before the initiation of any in-hospital treatment. However, pre-hospital antibiotic usage was not prospectively recorded. Partial data on antibiotic use were available only for the COVID-19 patients (n=145 across severity groups: 88- mild, 28- moderate, 14- severe, and 15- mortality group) and are provided in* ***Supplementary Table S1****.*”

**Discussion**: “*Interestingly, in our study, mild cases of COVID-19 exhibited the highest antibiotic exposure, with macrolides (22.7%), tetracyclines (14.1%), and beta-lactams (3.9%) being the most frequently prescribed. In contrast, severe and mortality groups showed lower overall antibiotic usage. Macrolides were administered to 20.8% of severe cases and 6.7% of mortality cases. Tetracyclines were more used in mild patients followed by severe (8.3%), moderate (6.1%) and mortality (0). Our clinical data indicate that β-lactam antibiotics were administered during treatment at varying frequencies (e.g., 3.9% in mild, 9.1 in moderate, 12.5% in severe, and 0% in mortality groups). While these data provide valuable insights into treatment patterns, they hold limited relevance to the ARG profiles detected, given the temporal disconnect between treatment and sample collection. It was worth noting that no patients in the mortality group received β-lactam and tetracyclines antibiotics. Despite the higher antibiotic exposure in the mild and moderate groups, resistance genes associated with cephalosporins, phenicols, and aminocoumarins, were predominantly detected in the severe and mortality groups. This apparent discrepancy highlights the complexity of ARG dynamics, suggesting that factors such as baseline resistance profiles, microbial ecology, and environmental exposures may play significant roles in ARG prevalence in severe cases.*”

**Limitations**: “*First, the lack of detailed antibiotic usage data prior to hospital admission restricts our ability to fully correlate resistome patterns with prior antimicrobial exposure. Antibiotic usage data were only partially available for COVID-19 patients and entirely unavailable for the dengue cohort, alongside missing information on hospitalization duration and comorbidities—factors that could influence microbial signatures.*”

- **Other clinical variables:**

Variables such as age, sex, and disease severity were collected for both cohorts and are already presented in the manuscript. To address the Reviewer’s comment, we have now added the following in the revised manuscript:

**Result 1:** “*In contrast, other severity-modulating factors, including age, gender, and comorbidities, were analyzed across both infections and their respective severity classes. Gender showed no significant differences across severity groups in either the COVID-19 or dengue cohorts. Age did not demonstrate significant differences across severity classes in the dengue cohort. However, in the COVID-19 cohort, age was significantly associated with severity when comparing all severity classes collectively. Notably, individual group comparisons, such as the moderate group (median age: 62) and the mortality group (median age: 65), revealed similar age distributions despite differing severity levels. This suggests that while age is associated with disease severity, it does not solely account for the observed differences in severity, indicating the influence of additional factors beyond age alone. Similarly, comorbidities among COVID-19 patients were not significantly associated with disease severity (Table 1). Comorbidity data for the dengue patients, however, were not available for analysis.*”

3. Detail on the antibiotic exposure, hospitalization durations, and ICU admission and comorbidities would enhance the findings of the study. At present, without this extra information, the direct linking of ARGs with viral disease does remain speculative. To improve, these factors should be accounted for.

We agree with the reviewer’s insightful comments that additional clinical details could enhance the findings of our study. In response to these suggestions, we have incorporated information regarding comorbidities, antibiotic exposure, and hospitalization durations into the updated manuscript. Below, we provide a detailed explanation of these updates and their implications.

While comprehensive data were limited for dengue patients, we were able to analyze comorbidity information for the COVID-19 cohort. The **updated clinical table** now includes details of the primary comorbidities observed, which include asthma, diabetes, hypertension, heart disease, thyroid disorders, and kidney-related conditions. Statistical analyses, including logistic regression models for each group (mild, moderate, severe, and mortality), revealed no significant associations between these comorbidities and the prevalence of antimicrobial resistance genes (ARGs) across disease severity, except in the mild group. In the mild group, a few genes showed significant but weak associations with certain comorbidities; however, these genes were not part of prevalent genes (*TEM-55, TEM-232, TEM-149,* and *TEM-192*) of COVID-19 mild which we have reported in our study, indicating that comorbidities likely did not significantly influence ARG patterns in this cohort.

**Updated Table 1:**

| **COVID-19** | **COVID-19 Clinical Parameters** | **Mild (n=97)** | **Moderate (n=95)** | **Severe (n=80)** | **Mortality(n=91 )** | **p-values** | |  |
| --- | --- | --- | --- | --- | --- | --- | --- | --- |
|  | Age | 46 | 62 | 58 | 65 | <0.0001 | | b |
|  | *SpO2* | 97 | 95 | 80 | 91.5 | <0.0001 | | b |
|  | *RdRp* | 23.12 | 28.57 | 27.46 | 18 | <0.0001 | | b |
|  | Hospital stay (days) | 6 | 12 | 10 | 9.5 | 0.0061 | | a |
|  | Gender F\|M | 54\|74 | 11\|22 | 9\|15 | 20\|39 | 0.64934 | | a |
| Comorbidities | Asthma | 1 (0.78) | 1 (3.03) | 0 | 0 | .298309 | | a |
|  | Diabetes | 15 (11.72) | 5 (15.15) | 6 (24) | 3 (5) | .907046 | | a |
|  | Heart disease | 3 (2.34) | 2 (6.06) | 1 (4) | 0 | .540653 | | a |
|  | Hypertension | 21 (16.41) | 8 (24.24) | 2 (8) | 4 (6.67) | .082804 | | a |
|  | Thyroid | 2 (1.56) | 0.00 | 2 (8) | 1 (1.67) | .12262 | | a |
|  | kidney | 5 (3.91) | 4 (12.12) | 0 | 0 | .067007 | | a |
|  | lungs | 2 (1.56) | 2 (6.06) | 0 | 0 | .138816 | | a |
|  | Others | 7 (5.47) | 4 (12.12) | 2 (8) | 2 (3.33) | .365068 | |  |
| **DENGUE** | **Dengue Clinical Parameters** | **Mild (n=45)** | **Moderate (n=46)** | **Severe (n=21)** |  | **p-values** | |  |
|  | Age | 27 | 26 | 35 |  | 0.13963 | | b |
|  | Gender F\|M | 15\|30 | 17\|29 | 4\|17 |  | 0.338071 | | a |
|  | NS1 antigen | 3.5 | 3.5 | 3.5 |  | 0.7536 | | b |
|  | **CBC** | | | | | | | |
|  | Total Leucocyte Count (TLC) | 5 | 3 | 2.7 |  | | < .001 | b |
|  | RBC Count | 4.8 | 4.835 | 4.78 |  | | 0.7556 | b |
|  | Packed Cell, Volume | 41.2 | 42.25 | 43.9 |  | | 0.184 | b |
|  | Platelet Count | 175 | 160 | 125 |  | | < .001 | b |
|  | Neutrophils | 75.9 | 63.3 | 62.6 |  | | < .001 | b |
|  | Lymphocytes | 14.1 | 24.5 | 26 |  | | < .001 | b |
|  | Monocytes | 9.8 | 10 | 9 |  | | 0.31923 | b |
|  | Eosinophils | 0.2 | 0.2 | 0.5 |  | | 0.32048 | b |
|  | Basophils | 0.5 | 0.6 | 0.5 |  | | 0.6906 | b |
|  | Hb | 13.6 | 13.9 | 14.1 |  | | 0.28362 | b |
|  | **LFT** | | | | | | | |
|  | Total Protein | 7.3 | 6.7 | 6.7 |  | | 0.02715 | b |
|  | Albumin | 4.4 | 4 | 4 |  | | 0.0449 | b |
|  | Globulin | 3.1 | 3 | 2.65 |  | | 0.0557 | b |
|  | A.G. ratio | 1.4 | 1.3 | 1.55 |  | | 0.18929 | b |
|  | Bilirubin (Total) | 0.7 | 0.4 | 0.75 |  | | 0.0184 | b |
|  | Bilirubin (Direct) | 0.135 | 0.09 | 0.175 |  | | 0.01765 | b |
|  | Bilirubin (Indirect) | 0.56 | 0.31 | 0.555 |  | | 0.0277 | b |
|  | SGOT- Aspartate Transaminase (AST) | 41 | 59 | 97.5 |  | | 0.0266 | b |
|  | SGPT- Alanine Transaminase (ALT) | 42 | 41 | 66 |  | | 0.1314 | b |
|  | AST/ALT Ratio | 0.93 | 1.48 | 1.705 |  | | 0.0767 | b |
|  | Alkaline Phosphatase | 67 | 80 | 82.5 |  | | 0.609 | b |
|  | GGTP (Gamma GT), Serum | 37 | 19 | 49 |  | | 0.0786 | b |

a-chi square, b-kruskal wallis

In the context of antibiotic exposures, it is essential to note that antibiotic administration occurred during the course of treatment, while sample collection was performed first thing upon hospital admission. This temporal separation suggests that the ARGs detected likely reflect pre-existing resistance profiles or community-acquired resistance rather than being directly influenced by the hospital-administered antibiotics. As per the reviewer’s suggestion, we have updated the clinical table to summarize the antibiotics administered to COVID-19 patients across disease severity groups. However, we acknowledge the absence of patient-level antibiotic treatment data for dengue patients.

Additionally, we have already given the hospitalization durations for COVID-19 patients in the clinical table. Unfortunately, similar data were unavailable for dengue patients, which we have highlighted as a limitation in the discussion section.

The following has been added to the revised manuscript:

**Result:** *“In the context of COVID-19, the presence of comorbidities prompted an investigation into potential associations between comorbidities and antimicrobial resistance genes (ARGs) across varying disease severities. Statistical analyses, including logistic regression models for each group (mild, moderate, severe, and mortality), revealed no significant associations between these comorbidities and the prevalence of ARGs across disease severity, except in the mild group. In the mild group, a few genes showed significant but weak associations with certain comorbidities; however, these genes were not part of prevalent genes (TEM-55, TEM-232, TEM-149, and TEM-192) of COVID-19 mild which we have mentioned above, indicating that comorbidities likely did not significantly influence ARG patterns in this cohort* ***(Supplementary Table S6)****.”*

4. There is evidence of possible sample bias in the study, where direct comparisons between clinical specimens derived from Covid19 patients (nasopharyngeal swabs) and dengue (blood samples) have been made; both have different microbiomes. It is currently unclear how sample origin affects resistome and microbiome composition, and thus this should be acknowledged in the text. Perhaps by using a sensitivity analysis where data is compared to type group only i.e. comparing results with dengue or covid19 cohorts respectively; or by testing for shared ARGs and taxa between both sample types.

We thank the reviewer for this insightful comment. We agree that comparing clinical specimens from different body sites—nasopharyngeal swabs for COVID-19 and blood for Dengue—can introduce potential variability due to the site-specific microbiome differences. Although, as mentioned before, the site specific sample collection was for the diseases studies, which are united by single stranded RNA viruses, causing infection.

To address this concern, we have now clarified in the revised manuscript that our primary aim was to characterise the transcriptionally active microbes and resistome at the relevant site of infection for each disease: the respiratory tract for COVID-19 and the bloodstream for Dengue. Additionally, to assess potential disease-specific microbial signatures independent of sample type, we performed a sensitivity analysis within the Dengue cohort, using an independent lab generated dataset. Towards addressing this suggestion, we included a dengue-negative control group comprising 20 febrile patients who tested NS1 antigen-negative, and whose blood total RNA samples were sequenced using the same protocol as the 112 dengue-positive patients. By comparing taxa derived from unmapped reads across both the groups and the difference confidence was calculated using the permanova test with 999 random permutations. Random permutations were controlled by stratifying the site origin type using strata argument within adonis2 function of vegan package in R, we observed distinct clustering, suggesting that the observed microbial differences are attributable to disease status rather than the sampling site **(Figure)**. This analysis reinforces the biological relevance of the disease-specific microbial profiles, while we still acknowledge that between-disease comparisons may be influenced by differing sample origins.

a.	TAMs abundance (Dengue positive and Dengue negative patients)

b.	ARGs abundance (Dengue positive and Dengue negative patients)

**Figure:** PCoA Plots representing distinct abundance clustering of (a) TAMs and (b) ARGs between dengue-positive and dengue-negative samples.

We have incorporated these clarifications and analyses into the revised manuscript as **Supplementary File S1**, titled “*Sensitivity Analysis Within the Dengue Cohort to Assess and Address Potential Sample Type Bias.*”

Additionally, the corresponding explanation has been added to the main text under **Results Section 4**, which reads: **“***While we acknowledge that comparing clinical specimens from different body sites—nasopharyngeal swabs for COVID-19 and blood for Dengue—can introduce potential variability due to the site-specific microbiome differences, our primary aim was to characterise the TAMs and resistome at the most clinically relevant site of infection for each disease: the respiratory tract for COVID-19 and the bloodstream for Dengue. To further evaluate whether the observed microbial and genes signatures were potential disease-specific microbial rather than artifacts of sampling type, we performed a sensitivity analysis within the Dengue cohort, using an independent, lab generated dataset. and observed distinct clustering, suggesting that the observed microbial differences are attributable to disease status rather than the sampling site* ***(Supplementary File S1)****. These findings reinforce the biological relevance of the identified microbial and ARGs profiles.***”**

At present much is inferred and claimed by the authors; but the above analyses would support more robust data interpretation.

**Part III – Minor Issues: Editorial and Data Presentation Modifications**
Reviewer #1:

5. There are minor errors in regards to grammar and fonts throughout the text. More citations could be used throughout to support claims and methods used. Abbreviations are stated without clarification which leads to poor readability in the text and in tables.

We thank the reviewer for highlighting these important presentation issues. In response, we have thoroughly proofread the manuscript to correct minor grammatical errors, ensure consistent font formatting throughout the text, to ensure that all abbreviations are clearly defined at first use and used consistently thereafter to improve readability.

Few examples: (red denotes update to the earlier version)

1. Result 1: For COVID-19, *SpO₂* (peripheral oxygen saturation) and *RdRp* gene (RNA-dependent RNA polymerase) were the key severity indicators.
2. Figure Legend 2: **(d)** Heatmaps displaying the differential abundance (log10 GCPM) of the top 30 abundant ARGs across the patient samples in **(i)** COVID-19, and **(ii)** dengue, where purple indicates lower gene abundance and yellow represents higher gene expression levels. The highlighted boxes display ARGs clustering based on abundance patterns: the top box (2d-i) shows ARGs uniformly high across Dengue samples, and the bottom box (2d-ii) shows those high across COVID-19. Other boxes represent ARGs that are abundant in a subset of patients but low in others, illustrating inter-patient variability in resistome profiles.
3. Result 3: Notably, genera such as *Escherichia*, *Klebsiella*, *Acinetobacter*, *Salmonella*, *Staphylococcus*, and *Enterococcus* are prominent carriers of ARGs,
4. Result 4: Of these, 20 ARGs were enriched in dengue, while only four—two aminoglycoside (*ANT(3'')-IIa*, *kdpE*) and two MDR (*TEM-1*, *TEM-243*)—were more abundant in COVID-19.

**Reviewer #2 -**

**Part I - Summary**

Reviewer #2: I was pleased to have the opportunity to review this manuscript. This manuscript uses a metatranscriptomic approach to explore the role of the resistome and microbiome in viral infections, focusing on COVID-19 and dengue patients. It was great to see additional attempts at metatranscriptomic characterisation, given limited work done in this area with regard to the microbiome.

The use of metatranscriptomics to infer active microbial contributions adds novelty, and the dual-disease comparison is commendable. However, the manuscript requires clarification of methodological definitions, more rigorous justification of analytical choices (e.g., thresholds, tool appropriateness), and moderation of some interpretations pending supporting metadata. I provide specific recommendations below, and look forward to seeing the updated manuscript.

We thank the Reviewer for the encouraging and constructive feedback along with suggestions to improve further. We're glad the novelty of our meta-transcriptomic approach and the dual-disease comparison were appreciated. In response to the comments, we have clarified methodological definitions, provided justification for analytical thresholds and tool choices, and moderated interpretations where supporting metadata were limited. These revisions have been incorporated throughout the revised manuscript to improve its clarity and rigor.

**Part II – Major Issues: Key Experiments Required for Acceptance**

Reviewer #2:

1. The manuscript makes strong claims about β-lactam antibiotic use driving ARG prevalence, particularly stating: "its consistently high abundance in both infections suggests strong selective pressure from widespread β-lactam antibiotic use..." This inference is unsupported without patient-level antibiotic treatment data. If such metadata exist, they should be incorporated into the analysis. If not, the language must be moderated to avoid overinterpretation, focusing instead on ARGs as a potential reservoir.

We thank the reviewer for this suggestion regarding the interpretation of β-lactam antibiotic use and ARG prevalence. While we acknowledge the absence of patient-level antibiotic treatment data for the dengue patients, we have incorporated data on antibiotic usage during the treatment of COVID-19 patients into the updated clinical table. In the context of antibiotic exposures, it is important to emphasize that sample collection was performed upon hospital admission, whereas antibiotics administered during the subsequent course of treatment **has been mentioned in Result 1.** This temporal separation strongly suggests that the ARGs detected in our study reflect pre-existing resistance profiles or community-acquired resistance rather than being directly influenced by hospital-administered antibiotics. Additionally, we acknowledge the absence of data on antibiotic usage prior to hospital admission for both groups, which limits the scope of our conclusions. **This limitation has been addressed in the “limitation” section.**

To address the reviewer’s suggestions, we have **added the supplementary table 1 in result 1** to include a summary of antibiotics administered during treatment for COVID-19 patients across the disease severity groups. Our clinical data indicate that β-lactam antibiotics were administered during treatment at varying frequencies **(e.g., 33.9% in mild, 9.1 in moderate,12.5% in severe, and 0% in mortality groups).** While these data provide valuable insights into treatment patterns, they hold limited relevance to the ARG profiles detected, given the temporal disconnect between treatment and sample collection. **We have mentioned this information in the discussion section in detail.**

In our study, the consistently high abundance of TEM-116, an extended-spectrum β-lactamase (ESBL), across both infections was observed in samples collected at the time of hospital admission. Since no antibiotics had been administered at this stage, the detected ARG profiles likely represent baseline resistance or community-acquired resistance patterns, potentially shaped by the external antibiotic pressures prior to hospitalization.

The following has been added in the revised manuscript:

**Supplementary Table 1: Antibiotic Data for COVID-19 Patients (n=145)**

| **S.No.** | **Antibiotics** | **COVID-19 Disease Severity (%)** | | | | **p-values** |
| --- | --- | --- | --- | --- | --- | --- |
|  |  | **Mild (n=128)** | **Moderate (n=33)** | **Severe (n=24)** | **Mortality(n=60)** |  |
| **1** | **Antiviral** | 47 (36.7) | 12 (36.4) | 9 (36) | 3 (5) | 0.000059 |
| **2** | **Anti-inflammatory** | 21 (16.4) | 2 (6.1) | 2 (8) | 5 (8.3) | 0.218311 |
| **3** | **Anticoagulant** | 16 (12.5) | 6 (18.2) | 0 | 0 | 0.396821 |
| **4** | **Antibacterial** | 53 (41.4) | 9 (27.3) | 9 (36) | 4 (6.7) | 0.000026 |
| **4A** | **Beta-lactams** | 5(3.9) | 3 (9.1) | 3(12.5) | 0 | 0.184581 |
| **4B** | **Tetracycline** | 18(14.1) | 2(6.1) | 2(8.3) | 0 | 0.379726 |
| **4C** | **Macrolides** | 29(22.7) | 6(18.2) | 5(20.8) | 4 (6.7) | 0.064477 |
| **4D** | **Nitro imidazole** | 0 | 1 (3) | 0 | 0 |  |
| **4E** | **Oxazolidinone** | 0 | 0 | 1(4.2) | 0 |  |

Additionally, we have now incorporated antibiotic-related information throughout the revised manuscript, including in the *Results*, *Discussion*, and *Limitations* sections, as follows:

**Result 1**: “*In our study, all samples from both the COVID-19 and dengue patient groups were collected on the first day of hospital reporting, prior to admission and before the initiation of any in-hospital treatment. However, pre-hospital antibiotic usage was not prospectively recorded. Partial data on antibiotic use were available only for the COVID-19 patients (n=145 across severity groups: 88- mild, 28- moderate, 14- severe, and 15- mortality group) and are provided in* ***Supplementary Table 1****.*”

**Discussion**: “*Interestingly, in our study, mild cases of COVID-19 exhibited the highest antibiotic exposure, with macrolides (22.7%), tetracyclines (14.1%), and beta-lactams (3.9%) being the most frequently prescribed. In contrast, severe and mortality groups showed lower overall antibiotic usage. Macrolides were administered to 20.8% of severe cases and 6.7% of mortality cases. Tetracyclines were more used in mild patients followed by severe (8.3%), moderate (6.1%) and mortality (0). Our clinical data indicate that β-lactam antibiotics were administered during treatment at varying frequencies (e.g., 3.9% in mild, 9.1 in moderate, 12.5% in severe, and 0% in mortality groups). While these data provide valuable insights into treatment patterns, they hold limited relevance to the ARG profiles detected, given the temporal disconnect between treatment and sample collection. It was worth noting that no patients in the mortality group received β-lactam and tetracyclines antibiotics. Despite the higher antibiotic exposure in the mild and moderate groups, resistance genes associated with cephalosporins, phenicols, and aminocoumarins, were predominantly detected in the severe and mortality groups. This apparent discrepancy highlights the complexity of ARG dynamics, suggesting that factors such as baseline resistance profiles, microbial ecology, and environmental exposures may play significant roles in ARG prevalence in severe cases.*”

**Limitations**: “*First, the lack of detailed antibiotic usage data prior to hospital admission restricts our ability to fully correlate resistome patterns with prior antimicrobial exposure. Antibiotic usage data were only partially available for COVID-19 patients and entirely unavailable for the dengue cohort, alongside missing information on hospitalization duration and comorbidities—factors that could influence microbial signatures.*”

**Result 2:** “*Interestingly, TEM-116 was consistently abundant in both infections, samples collected at the point of hospital admission. As no antibiotics had been administered before this stage, the observed ARG profiles are likely indicative of baseline or community-acquired resistance, influenced by prior exposure to environmental antibiotic pressures.*”

Therefore, as per the reviewer’s suggestion regarding over-interpretation, we have revised the manuscript language to moderate the inference about selective pressure from the β-lactam antibiotic use. Instead of attributing ARG prevalence directly to widespread β-lactam use, we now present the finding as evidence of ARGs serving as a potential reservoir shaped by broader environmental or community-level selective pressures.

The revised statement reads:

*"It’s consistently high abundance in both infections suggests the presence of pre-existing reservoirs shaped by external antibiotic pressures, underscoring the role of ARGs as a potential reservoir for resistance."*

2. The low threshold used for filtering ARGs (30% gene coverage and presence in at least 10% of samples) may reduce the reliability of the findings. The authors should provide a breakdown of how many ARGs surpass higher, more confident thresholds (e.g., ≥70% coverage), and discuss how the current threshold affects specificity.

We acknowledge the reviewer’s concern regarding the relatively low threshold used for ARG detection (30% gene coverage and presence in at least 10% of samples). Our rationale for using this threshold was to avoid prematurely excluding potentially relevant ARGs, especially considering the fragmented nature of RNA-seq data and the variability in the gene expression levels across samples.

Still, taking into account the reviewer suggestion, we have now provided a breakdown of the numbers of ARGs that surpassed various thresholds starting from 30% to 90% coverage. Here’s the graph depicting the same:

Additionally, to address this concern, we further stratified the detected ARGs into two categories: those with **30–50% gene coverage** and those with **>50% gene coverage**. We observed that the majority of ARGs with >50% coverage were also detected in the 30–50% category, indicating consistency in the detection of highly confident genes. Dengue has similar numbers of genes across 30-90% coverage, however COVID-19 shows some genes that are found <50% coverage.

For ARGs found only within the 30–50% coverage group (i.e., not meeting the >50% threshold), which are 92 and 14 in COVID-19 and Dengue respectively, we examined their coverage profiles in greater detail. We found that many of these genes exhibited RNA-seq reads mapping to **multiple, non-contiguous regions** across the gene loci, including both the **start, middle and end regions** of the gene. This multi-region coverage pattern vis-à-vis gene adds confidence that the partial coverage was not due to random or spurious mapping, but likely represents genuine transcriptional activity. In the COVID-19 samples, 40 genes predominantly exhibited multisite occurrences, while the remaining 52 genes were more frequently associated with single-site occurrences. In contrast, only 5 genes in dengue samples showed a predominance of multisite presence, as illustrated in the accompanying bar plots.

Therefore, while a higher threshold like ≥70% could indeed increase specificity, it may also result in the loss of biologically relevant signals. Our approach balances sensitivity and specificity by including ARGs above 30% coverage while interpreting genes in the 30–50% range with additional scrutiny based on their coverage patterns.

We have incorporated these clarifications and analyses into the revised manuscript as **Supplementary File S7**, titled “*Distribution of Microbial ARG Coverage and Multi-/Single-Site ARGs Within the 30–50% Threshold Range.*”

Additionally, the corresponding explanation has been added to the main text under **Methods section**, which reads: **“***This threshold balances sensitivity and specificity, accounting for RNA-seq data fragmentation and expression variability. We stratified ARGs into 30–50% and >50% coverage groups, observing that most high-coverage ARGs were also detected in the lower range, indicating consistent detection. Dengue samples showed stable ARG counts across 30–90% coverage, while COVID-19 had several ARGs detected only below 50%. To enhance resolution, we have now provided a detailed breakdown of ARGs surpassing coverage thresholds from 30% to 90%, along with a graphical summary in the* ***Supplementary File S****7.***”**

3. The authors should consider mapping their sequencing reads to the Dengue virus and SARS-CoV-2 reference genomes to verify the presence of the primary pathogens. This serves as an important quality control measure to confirm that sequencing successfully captures host-pathogen dynamics and supports conclusions based on microbial transcriptional activity.

Thank you for the insightful suggestion. We would like to clarify that we had indeed mapped the sequencing reads to the Dengue virus and SARS-CoV-2 reference genomes as a validation but did not mention explicitly in the original submission, which we have now explicitly mentioned in the revised manuscript. Our analysis confirmed the presence of viral reads in the respective samples, supporting the presence of the primary pathogens. We have attached coverage of viral reads in both dengue and COVID-19 patients’ samples to strengthen our findings.

Specifically, dengue virus reads were consistently detected in the 112 dengue-positive samples, with genome coverage ranging from 17% to 100%. Notably, 72 of these samples exhibited high dengue virus genome coverage (>90%) **(Figure a)**.

For the 251 COVID-19 patient samples, a significant amount of SARS-CoV-2 reads were detected in 213 samples ranging between 20-100% with 163 samples having more than 50% viral reads **(Figure b)**.

a.

b.

It is important to note that since both viruses possess positive-sense single-stranded RNA (ssRNA) genomes, we observed substantial alignment of viral RNA reads to their respective reference genomes. This indicates effective capture of microbial sequences in our meta-transcriptomic analysis. However, unlike RNA viruses, the bacteria detected in our study are typically composed of DNA, and what we capture through meta-transcriptomics (RNA) reflects only the actively transcribed portion of the genome rather than the entire genome. As a result, while bacterial transcripts mapped back to their corresponding species, the genome coverage is inherently partial and not expected to reach 100%. These findings confirm that our total RNA sequencing data captured the primary viral pathogens, supporting confident downstream analyses of the microbial transcriptomic profiles.

The same has now been incorporated into the revised manuscript as **Supplementary File S4**, titled “*Genome Coverage of Dengue Virus and SARS-CoV-2 Across Patient Samples.*”

Additionally, the corresponding explanation has been added to the main text under **Methods section**, which reads: **“***Additionally, to confirm the presence of primary pathogens, sequencing reads were mapped to the reference genomes of Dengue virus and SARS-CoV-2. Dengue virus reads were consistently detected in all 112 dengue-positive samples, with genome coverage ranging from 17% to 100%. In the COVID-19 cohort, SARS-CoV-2 reads were detected in 213 samples, with coverage ranging from 20% to 100%* ***(Supplementary File S4)****.***”**

4. The use of Bracken for metatranscriptomic analysis should be justified more robustly or replaced. Bracken's assumptions of proportionality between read counts and species abundance are more appropriate for DNA-based metagenomics. Its use here may introduce systematic biases.

We thank the reviewer for this important comment. While we acknowledge that Bracken was originally developed for DNA-based metagenomic datasets and its assumptions are most suited to DNA abundance estimation, we chose to use the Kraken2-Bracken pipeline due to its demonstrated performance and accuracy in large-scale metagenomic studies, including those involving low-quality or complex read data.

Recent studies have validated the robustness of the Kraken/Bracken pipeline for species-level classification even under suboptimal conditions. For instance, a benchmarking study (PMID: 36553546, https://doi.org/10.3390/genes13122280) showed that Kraken2/Bracken maintained high classification accuracy even with low-quality reads using synthetic datasets. Another study (PMID: 38425025, https://doi.org/10.1080/19490976.2024.2323235) demonstrated that Bracken significantly improves species-level abundance estimates by probabilistically reallocating reads from higher taxonomic levels, thus refining Kraken2's initial classifications and improving the reliability of downstream analyses.

Further, for this study, we explored various methods for analyzing relative abundance, as shown in Figure, including: (a) using raw and normalized read counts directly from Bracken, (b) manually calculating relative abundance from the raw reads, and (c) using normalized read counts. In all cases, the microbial distribution patterns remained consistent, with the same top taxa identified across methods. This consistency suggests that the relative abundance estimates are robust to the choice of calculation approach.

The corresponding explanation has been added in the revised manuscript as **Supplementary File S5** (*Evaluation of Bracken-Derived TAMs Relative Abundance Estimates Using Multiple Calculation Methods.*) under **Methods section**, which reads: **“***Although Bracken was originally designed for DNA-based metagenomic data, we used the Kraken2-Bracken pipeline due to its proven accuracy and robustness, even with low-quality or complex read datasets. Benchmarking studies (https://doi.org/10.3390/genes13122280, https://doi.org/10.1080/19490976.2024.2323235) have validated Kraken2/Bracken’s high classification accuracy and its ability to refine species-level abundance estimates by probabilistically reallocating reads from higher taxonomic levels. To ensure the robustness of our analysis, we evaluated multiple approaches for calculating relative abundance—including direct use of Bracken raw and normalized counts, and manual normalization. Across all methods, the microbial distribution patterns and top taxa remained consistent, indicating that the relative abundance estimates are stable regardless of the calculation strategy* ***(Supplementary File S5)****.***”**

5. There is inconsistency and ambiguity in the use of the terms non-canonical metatranscriptomics and holo-transcriptomics. The manuscript must clearly define both terms and clarify whether they are interchangeable or distinct, and ensure consistent use throughout.

We agree that the use of both “non-canonical metatranscriptomics” and “holo-transcriptomics” may have caused confusion. In the revised manuscript, we have removed the term “holo-transcriptomics” to maintain clarity and consistency. We now exclusively use the term “non-canonical metatranscriptomics”. We agree that the term **“non-canonical metatranscriptomics”** was not clearly defined in the original manuscript. In the revised version, we have now explicitly clarified this term and the rationale behind our approach and added in the **Introduction section**:

- **Term clarification**: By “non-canonical metatranscriptomics,” we refer to the repurposing of host-transcriptome total RNA-seq data—originally generated for host transcriptomics studies— to identify and quantify transcriptionally active microbes (TAMs) in this study. Unlike traditional metatranscriptomic approaches, which involve RNA extraction from isolated microbial communities and targeted microbial enrichment, our method starts with total RNA extracted from clinical human samples and applies computational filtering to remove host-aligned reads post-sequencing. Reason we believe it is better way to capture the host and microbial interactome is because we are doing no preferential enrichment or depletion for either human host or microbial communities, but capturing the natural dynamics.

In the Introduction section, we have added:

*“In this study, we employed a previously lab used “non-canonical meta-transcriptomics” approach, which we define as the repurposing of host total RNA-seq data—originally generated for the host transcriptomics—to identify and quantify transcriptionally active microbes (TAMs). Unlike conventional metagenomics or metatranscriptomics, which involve microbial DNA/RNA enrichment, our method starts from native clinical samples based on disease manifestation site (e.g., blood or nasopharyngeal swabs for Dengue and COVID-19) and removes host-aligned reads computationally to analyze microbial expression. This strategy enables unbiased profiling of active microbial communities without introducing potential experimental biases from the enrichment protocols, which can distort transcript abundance in low-biomass, host-dominated samples.”*

**Part III – Minor Issues: Editorial and Data Presentation Modifications**
Reviewer #2:

6. The statement in the Introduction: "The overuse of antibiotics, both in clinical settings and beyond, accelerates antimicrobial resistance (AMR), a crisis projected to cause 10 million deaths annually by 2050.” requires citation

Thank you for bringing the inadvertent missing reference to our attention. We have included the citation in the revised version of the paper.

*Here is the revised version of the portion from paper*

The overuse of antibiotics, both in clinical settings and beyond, accelerates antimicrobial

resistance (AMR), a crisis projected to cause 10 million deaths annually by 2050 (10.1016/S0140-6736(24)01867-1).

7. Genus and species names (e.g., Escherichia, Klebsiella) are not italicized consistently across the manuscript. This should be corrected in all sections, including the Conclusions.

We have now thoroughly reviewed the manuscript and corrected all instances where genus and species names were not italicized, ensuring consistent and proper formatting throughout the manuscript.

8. The sentence "Building on our previous research on TAMs…” is vague. Please specify the findings from prior work and cite appropriately to provide context.

We sincerely thank the reviewer for this suggestion. We have now revised the sentence and it can be read as follows:

“*Building on our previous research on TAMs, that linked severe dengue to greater microbial diversity, enrichment of opportunistic species like Bacillus cereus and Burkholderia pseudomallei, and elevated microbial energy metabolism, we also reported variant-specific shifts in TAMs during SARS-CoV-2 infection—highlighting Acinetobacter baumannii and Pseudomonas aeuroginosa as a potential biomarker associated with immune response and clinical severity in Delta VOC [10,12]. We now expand this investigation, employingthis study employs a non-canonical meta-transcriptomics approach to identify and compare the resistome and microbiome profiles of COVID-19 and dengue patients, providing a new perspective of AMR evolution in infectious disease settings.*”

9. Inconsistent terminology for TAMs is used: "transcriptionally active microbial hosts” in the abstract and "metabolically or transcriptionally active ones” in the Introduction. These should be harmonized.

We sincerely thank the reviewer for highlighting the inconsistent terminology used for transcriptionally active microbes (TAMs). We acknowledge that the use of "transcriptionally active microbial hosts" in the abstract and "metabolically or transcriptionally active ones" in the introduction may have caused confusion. To address this, we have revised the manuscript to consistently use the term "***transcriptionally active microbes"*** throughout, including in the abstract, introduction, and all subsequent sections.

10. Some methodological content appears in the Results section. Please ensure all analytical steps and parameter descriptions are placed in the Methods and not repeated in Results unless essential for interpretation.

We thank the reviewer for this valuable observation. In response, we have carefully revised the manuscript to ensure that all analytical procedures, parameter settings, and other methodological descriptions have been moved to the Methods section, where appropriate.

11. Figure 2D is difficult to interpret. The heatmaps appear split (e.g., 2D-i) with boxed genes and no explanation for separation or grouping. Labeling must be clarified, and the figure legend should explain how heat maps are structured and what the boxes denote.

We appreciate the reviewer’s feedback regarding Figure 2D. To improve clarity, we have revised the figure to enhance labeling and better delineate the structure of the heatmaps.

The following has now been added to the figure legend: “*The highlighted boxes display ARGs clustering based on abundance patterns: the top box (2d-i) shows ARGs uniformly high across Dengue samples, and the bottom box (2d-ii) shows those high across COVID-19. Other boxes represent ARGs that are abundant in a subset of patients but low in others, illustrating inter-patient variability in resistome profiles*.”

The updated figures are also attached below for your reference.

12. The statement: "847 and 40 ARGs exclusive to COVID-19 and dengue..." lacks sufficient detail on detection confidence. This is partly addressed in Part II but should also be reflected in the figure or text to guide reader interpretation.

We appreciate the reviewer’s comment regarding the need for clarity on detection confidence for the ARGs reported as exclusive to COVID-19 and dengue. In response, we have revised the text to specify that the identification of 847 and 40 ARGs exclusive to COVID-19 and dengue, respectively, was based on unfiltered detection across the study samples. To address confidence in ARG presence, we later applied more stringent criteria, retaining only those ARGs with ≥30% gene coverage and presence in at least 10% of the patient samples, to define the dominant and most abundant ARGs (as detailed in Part II of the Results).

The text now read as: “*Notably, 457 ARGs were shared between both the infections, with 847 and 40 ARGs exclusive to COVID-19 and dengue respectively. The identification of 847 and 40 ARGs exclusive to COVID-19 and dengue, respectively, was based on unfiltered detection across the study samples.*”

13. Formatting of software tools is inconsistent. FastQC is mentioned without a version, while Trimmomatic includes a version. Please align formatting for clarity.

Thank you for this helpful observation. We agree that consistent formatting of software tools improves clarity and reproducibility. We have now included the version number for FastQC and reviewed the manuscript to ensure consistent formatting for all mentioned tools.

Revised sentence in manuscript:

*“Quality assessment was performed using FastQC (v0.11.9), and adapter trimming and low-quality read filtering were carried out using Trimmomatic (v0.39). Human genome sequences were removed by aligning reads to the reference genome (Ensembl hg38) using HISAT2 (v2.2.1)”.*

14. The Methods section mentions the use of QIAGEN kits with "specific adjustments, including..." but does not list all modifications. Either enumerate all adjustments or revise the sentence to avoid implying there are unlisted changes.

Thank you for this helpful suggestion. We have revised the Methods section to clearly enumerate all modifications made to the standard QIAGEN kit protocol.

*Below is the revised version*

*“RNA extraction from these samples was conducted using the QIAGEN QIAamp RNA Blood Mini Kit with specific protocol modifications to optimize yield and quality for library preparation. Incubation and centrifugation durations during the erythrocyte lysis step were reduced by 5 minutes to minimize RNA degradation and prevent excessive cell disruption, which can release contaminants. Additionally, a 2–3 minute incubation was introduced during all washing steps to enhance RNA purity by allowing more effective removal of residual proteins, salts, and other impurities that can interfere with library preparation and sequencing.”*

15. The thresholds used to define TAMs (e.g., >0.1% for species) are relatively low. Though this point is related to method justification, a brief rationale should be added or referenced to support their choice.

We thank the reviewer for highlighting the need to justify the TAMs threshold. We have added a brief rationale in the Methods section, stating that this threshold is widely supported for identifying biologically relevant species while minimizing spurious detections (10.1016/j.marenvres.2025.107169, 10.1186/s12915-014-0087-z, 10.1038/nature23889). We appreciate this suggestion, which enhances the manuscript’s clarity.

The threshold of >0.1% relative abundance for defining TAMs was selected based on our goal to identify species that make a measurable contribution within their respective phyla, even if their individual abundance may appear modest. To support this, we systematically evaluated the contribution of these species (with >0.1% relative abundance) to the total abundance of their corresponding phyla.

Specifically, we summed the total number of all species within each phylum that met the >0.1% criterion. Our analysis revealed that these species, despite the threshold appearing low in absolute terms, collectively account for a substantial portion of the phylum’s total abundance—often representing the dominant constituents of that phylum in a given sample.

| **COVID-19 Abundant Phyla** | **Species with >0.1% RA in 50% of sample** | **Total species in phyla** | **Counts Percentage** |
| --- | --- | --- | --- |
| Actinobacteria | 11 | 610 | 1.8 |
| Bacteroidetes | 7 | 285 | 2.5 |
| Firmicutes | 24 | 628 | 3.8 |
| Fusobacteria | 3 | 17 | 17.6 |
| Negarnaviricota | 2 | 215 | 0.9 |
| Proteobacteria | 30 | 1806 | 1.7 |
| Tenericutes | 3 | 95 | 3.2 |

| **Dengue Abundant Phyla** | **Species with >0.1% RA in 50% of sample** | **Total species in phyla** | **Counts Percentage** |
| --- | --- | --- | --- |
| Actinobacteria | 17 | 608 | 2.8 |
| Artverviricota | 1 | 39 | 2.6 |
| Bacteroidetes | 3 | 285 | 1.1 |
| Cyanobacteria | 4 | 122 | 3.3 |
| Duplornaviricota | 1 | 25 | 4.0 |
| Euryarchaeota | 3 | 170 | 1.8 |
| Firmicutes | 16 | 625 | 2.6 |
| Kitrinoviricota | 2 | 109 | 1.8 |
| Negarnaviricota | 2 | 128 | 1.6 |
| Nucleocytoviricota | 1 | 105 | 1.0 |
| Peploviricota | 5 | 83 | 6.0 |
| Pisuviricota | 1 | 155 | 0.6 |
| Planctomycetes | 1 | 17 | 5.9 |
| Proteobacteria | 48 | 1795 | 2.7 |
| Spirochaetes | 1 | 48 | 2.1 |
| Tenericutes | 10 | 95 | 10.5 |
| NA | 5 | 367 | 1.4 |

We have incorporated the method justification into the revised manuscript as **Supplementary File S6**, titled “*Cumulative Contribution of TAMs with >0.1% Relative Abundance to the Total Abundance Within the Corresponding Phyla.*”

Additionally, the corresponding explanation has been added to the main text under **Methods Section**, which reads: **“***The >0.1% relative abundance cutoff is commonly used in microbiome studies to balance sensitivity while minimizing noise from low-abundance taxa [36–38]. This threshold also aligns with our objective to capture species that make a meaningful contribution within their respective phyla, even at modest absolute abundance. To validate this, we systematically evaluated the cumulative contribution of >0.1% abundant species to the total abundance of their corresponding phyla* ***(Supplementary File S6)****.***”**

16. The claim: "this consistency reinforces the idea that while ARGs play a crucial role in COVID-19 severity, they have minimal impact on dengue progression” could be misleading. It is also possible that COVID-19 patients had more antibiotic exposure. This point is acknowledged later in the manuscript, but it should be flagged here as an interpretative limitation.

Thank you for this valuable observation. We agree that the original statement may have overstated the interpretation and did not sufficiently account for potential confounding factors, such as differential antibiotic exposure between COVID-19 and dengue patients. We have revised the sentence to more cautiously reflect the findings and now explicitly acknowledge the possibility that increased ARG presence in COVID-19 patients may be influenced by higher rates of antibiotic usage.

The revised sentence could be read as: “*This consistency reinforces the idea that ARGs play a crucial role in COVID-19 severity. While this could be influenced by prior antibiotic use, as mentioned before our samples were collected on the first day of hospital reporting, reducing the likelihood that elevated ARG levels are solely due to in-hospital antibiotic administration. However, increased antibiotic use before hospital admission may still be a contributing factor*.”

17. The final paragraph of the Conclusions could benefit from a brief note on limitations (e.g., lack of metadata, challenges of functional inference from RNA data) and a comment on the applicability of this method to public health surveillance (cost, scalability).

We thank the reviewer for this valuable suggestion. In response, we have separated the Conclusions and Limitations into distinct sections with clear headers.

The updated Limitations section reads as follows:

*“While our study provides detailed and important insights into the resistome and active microbial communities in the COVID-19 and dengue patients, there are a few limitations that should be addressed in future work to enhance interpretability and clinical relevance. First, the lack of detailed antibiotic usage data prior to hospital admission restricts our ability to fully correlate resistome patterns with prior antimicrobial exposure. Antibiotic usage data were only partially available for COVID-19 patients and entirely unavailable for the dengue cohort, alongside missing information on hospitalization duration and comorbidities—factors that could influence microbial signatures. Another potential limitation is the absence of dietary information, which could be a confounding factor; however, current evidence indicates that diet has minimal direct influence on the blood and nasal microbiomes. Additionally, meta-transcriptomics, while powerful in capturing active microbial and functional gene expression, reflects RNA-level dynamics, which may not always correspond to downstream protein activity. Nonetheless, transcriptomic profiles serve as a reliable surrogate for assessing real-time microbial functional dynamics, particularly in the absence of protein-level data. Future integration of complementary methods such as metaproteomics could further refine functional interpretations, but the current findings already provide meaningful and biologically relevant insights. We also acknowledge the absence of healthy controls and extraction-negative controls in the current design. However, our objective was to compare disease-specific microbiome and resistome profiles between two infectious diseases. We plan to incorporate both extraction blanks and dengue-negative febrile illness controls in future work that will expand on this study by exploring severe and non-severe dengue cases. Finally, all patient samples in this study were collected from North India, which may limit the generalizability of our findings to other geographic regions with differing healthcare infrastructure and antibiotic usage patterns. Moreover, while this meta-transcriptomic approach offers valuable insights, its broader adoption in public health surveillance remains constrained by cost, complexity, and infrastructure—particularly in low- and middle-income countries (LMICs). Streamlining protocols and developing targeted transcriptomic panels may help increase feasibility for routine surveillance and outbreak preparedness. Further research is needed to unravel these complexities and their implications for AMR management in viral infections.”*

**Reviewer #3 -**

**Part I - Summary**

Reviewer #3: Study explores microbiome/resistome links to dengue and COVID-19 (with a range of clinical outcomes), an important and overlooked area, using “non-canonical meta-transcriptomics” (looking at transcriptionally active microbes?). A non-overlapping cohort of COVID/dengue was used, and different samples assayed (NP vs blood), from which RNAseq was carried out and analysed for ARGs by class etc (and later to bacterial species), and linked to clinical outcomes/compared between infections. This is important descriptive data that highlights some interesting findings with potentially high relevance to clinical management and our understanding of the pathogenesis of viral diseases and their biology. From this one study, a lot of data are generated and many analyses are presented. However, serious questions remain about the study.

We sincerely thank the Reviewer for acknowledging the novelty and relevance of our study, as well as the potential clinical and biological implications of our findings. We also acknowledge the constructive suggestions and agree that such an integrative approach—spanning multiple cohorts, sample types, and analytical layers—requires careful contextualization. We hope the revised version addresses these concerns and reinforces the strength and reproducibility of our findings.

**Part II – Major Issues: Key Experiments Required for Acceptance**
Reviewer #3:

1. The biggest one is that this is a cross-sectional study on two non-overlapping cohorts. Ideally, results would be validated in an independent cohort at least.

We acknowledge that our study is cross-sectional and based on two non-overlapping cohorts. While this design allowed us to characterize and compare the microbiome and resistome profiles at the site of infection in COVID-19 and dengue patients, we agree with the reviewer that validation in an independent cohort would strengthen the generalizability of our findings. To address this, although we planned follow-up studies that include additional cohorts, including dengue-negative and varying severity groups, to validate and extend these observations, we present the findings over here wherein **we analysed a separate in-house study** involving an independent cohort of 58 dengue patients, of whom 26 had severe dengue and 32 had non-severe dengue, categorized according to WHO guidelines. This cohort also included dengue-negative patients presenting with febrile illness but testing negative for NS1 antigen. While this is maturing as a separate study to explore/elucidate functional microbiome and resistome differences vis-à-vis differential dengue severity, we have now used our unpublished work to support the reproducibility of our current findings.

Specifically, we compared antimicrobial resistance genes (ARGs) between the discovery (n=112) and validation (n=58) cohorts and identified 300 total ARGs, with 208 shared across both the cohorts **(Figure 1a)**. We further examined abundant shared ARGs (≥30% coverage in ≥10% of samples) and transcriptionally active microbes (TAMs) (≥0.1% relative abundance in ≥50% of samples), applying the same thresholds used in the current study. The analysis showed strong concordance between cohorts, with a high correlation for ARG abundance (R² = 0.93, p = 0.0067; **Figure 1b**) and microbial composition (R² = 0.77, p < 2.2 × 10⁻¹⁶; **Figure 1c**). These results support the reproducibility and robustness of our approach across independent patient groups.

We have now added a part of this analysis in the **Result section** which reads as “*To validate and extend our findings, we analyzed data from an independent in-house cohort of 58 dengue patients (26 severe, 32 non-severe; classified per WHO guidelines), along with dengue-negative febrile controls (NS1-negative). We identified 300 ARGs across the discovery (n=112) and validation (n=58) cohorts, with 208 shared between them. Using the same thresholds—≥30% coverage in ≥10% of samples for ARGs and ≥0.1% relative abundance in ≥50% of samples for TAMs—we observed strong concordance between cohorts. ARG abundance was highly correlated (R² = 0.93, p = 0.0067), as was microbial composition (R² = 0.77, p < 2.2 × 10⁻¹⁶), supporting the robustness and reproducibility of our approach across independent patient groups* ***(Supplementary File S3)****.*”

This is unpublished data from an ongoing study. For transparency and reference, we have now uploaded the dataset to NCBI under the SRA ID: PRJNA1279769, and have included this information in the Data Availability section of the manuscript, and provided a new **Supplementary File S3** titled “*Comparative analysis for concordance of ARGs and microbial abundance between two independent dengue cohorts for validation of study findings*.” with detailed description. We are also providing the reviewer’s access link for your convenience: <https://dataview.ncbi.nlm.nih.gov/object/PRJNA1279769?reviewer=hr34asdgjbr09rrthofj0uua8k>

2. There are issues with the description of their study, namely antibiotic usage patterns between the different diseases and severities. Do the authors have this? Similarly, were any patients co-infected with dengue and COVID?

We appreciate the reviewer’s valuable comments regarding the description of antibiotic usage patterns and co-infections. Below, we provide clarification on these points:

**Antibiotic Usage Patterns Across Diseases and Severities:**

We acknowledge the importance of antibiotic exposure as a potential confounding factor in AMR analyses. To augment clarification vis-à-vis our study, all samples were collected on the first day of hospital reporting—prior to admission and before initiation of any in-hospital treatment. Therefore, antibiotic administration within the hospital setting did not influence the samples used for meta-transcriptomic analysis. However, since we did not prospectively capture patients’ pre-hospital antibiotic use history (i.e., antibiotics taken before arriving at the hospital), this remains a limitation for a subset of cases. Given that our AMR findings are based on first-contact, treatment-naïve samples, we believe the immediate impact of antibiotics on the observed resistome is minimal.

Initially, we excluded this variable due to incomplete data across both the cohorts. While partial antibiotic usage data were available for COVID-19 patients, these data were not collected for dengue patients.

To address the reviewer’s query, we have now added the available antibiotic information for COVID-19 patients as a **“Supplementary Table S1”** in the revised manuscript, and is placed here for reference.

**Supplementary Table S1: Antibiotic Data for COVID-19 Patients (n=145)**

| **S.No.** | **Antibiotics** | **COVID-19 Disease Severity (%)** | | | | **p-values** |
| --- | --- | --- | --- | --- | --- | --- |
|  |  | **Mild (n=128)** | **Moderate (n=33)** | **Severe (n=24)** | **Mortality(n=60)** |  |
| **1** | **Antiviral** | 47 (36.7) | 12 (36.4) | 9 (36) | 3 (5) | 0.000059 |
| **2** | **Anti-inflammatory** | 21 (16.4) | 2 (6.1) | 2 (8) | 5 (8.3) | 0.218311 |
| **3** | **Anticoagulant** | 16 (12.5) | 6 (18.2) | 0 | 0 | 0.396821 |
| **4** | **Antibacterial** | 53 (41.4) | 9 (27.3) | 9 (36) | 4 (6.7) | 0.000026 |
| **4A** | **Beta-lactams** | 5(3.9) | 3 (9.1) | 3(12.5) | 0 | 0.184581 |
| **4B** | **Tetracycline** | 18(14.1) | 2(6.1) | 2(8.3) | 0 | 0.379726 |
| **4C** | **Macrolides** | 29(22.7) | 6(18.2) | 5(20.8) | 4 (6.7) | 0.064477 |
| **4D** | **Nitro imidazole** | 0 | 1 (3) | 0 | 0 |  |
| **4E** | **Oxazolidinone** | 0 | 0 | 1(4.2) | 0 |  |

Additionally, we have now incorporated antibiotic-related information throughout the revised manuscript, including in the *Results*, *Discussion*, and *Limitations* sections, as follows:

**Result 1**: “*In our study, all samples from both the COVID-19 and dengue patient groups were collected on the first day of hospital reporting, prior to admission and before the initiation of any in-hospital treatment. However, pre-hospital antibiotic usage was not prospectively recorded. Partial data on antibiotic use were available only for the COVID-19 patients (n=145 across severity groups: 88- mild, 28- moderate, 14- severe, and 15- mortality group) and are provided in* ***Supplementary Table S1****.*”

**Discussion**: “*Interestingly, in our study, mild cases of COVID-19 exhibited the highest antibiotic exposure, with macrolides (22.7%), tetracyclines (14.1%), and beta-lactams (3.9%) being the most frequently prescribed. In contrast, severe and mortality groups showed lower overall antibiotic usage. Macrolides were administered to 20.8% of severe cases and 6.7% of mortality cases. Tetracyclines were more used in mild patients followed by severe (8.3%), moderate (6.1%) and mortality (0). Our clinical data indicate that β-lactam antibiotics were administered during treatment at varying frequencies (e.g., 3.9% in mild, 9.1 in moderate, 12.5% in severe, and 0% in mortality groups). While these data provide valuable insights into treatment patterns, they hold limited relevance to the ARG profiles detected, given the temporal disconnect between treatment and sample collection. It was worth noting that no patients in the mortality group received β-lactam and tetracyclines antibiotics. Despite the higher antibiotic exposure in the mild and moderate groups, resistance genes associated with cephalosporins, phenicols, and aminocoumarins, were predominantly detected in the severe and mortality groups. This apparent discrepancy highlights the complexity of ARG dynamics, suggesting that factors such as baseline resistance profiles, microbial ecology, and environmental exposures may play significant roles in ARG prevalence in severe cases.*”

**Limitations**: “*First, the lack of detailed antibiotic usage data prior to hospital admission restricts our ability to fully correlate resistome patterns with prior antimicrobial exposure. Antibiotic usage data were only partially available for COVID-19 patients and entirely unavailable for the dengue cohort, alongside missing information on hospitalization duration and comorbidities—factors that could influence microbial signatures.*”

**Co-infection Status:**

In our study, none of the patients were clinically reported to be co-infected with both SARS-CoV-2 and dengue virus.

3. There is limited information on controls. The authors need to show data on negative controls for extraction and kits. Similarly, no healthy controls are assayed from relevant tissues.

We thank the reviewer for highlighting the importance of appropriate controls in meta-transcriptomic studies. As noted, our current study was specifically designed to compare the microbiome and resistome between two RNA viral diseases—COVID-19 and dengue—and across clinical differential severity subgroups. As such, healthy controls and extraction-negative controls were not included in this initial study design. We acknowledge this as a limitation and have explicitly discussed it in the revised *Limitations* section of the manuscript.

The updated Limitations section reads as follows which partially captures this aspect:

“*While our study provides detailed and important insights into the resistome and active microbial communities in the COVID-19 and dengue patients, there are a few limitations that should be addressed in future work to enhance interpretability and clinical relevance. First, the lack of detailed antibiotic usage data prior to hospital admission restricts our ability to fully correlate resistome patterns with prior antimicrobial exposure. Antibiotic usage data were only partially available for COVID-19 patients and entirely unavailable for the dengue cohort, alongside missing information on hospitalization duration and comorbidities—factors that could influence microbial signatures. Another potential limitation is the absence of dietary information, which could be a confounding factor; however, current evidence indicates that diet has minimal direct influence on the blood and nasal microbiomes. Additionally, meta-transcriptomics, while powerful in capturing active microbial and functional gene expression, reflects RNA-level dynamics, which may not always correspond to downstream protein activity. Nonetheless, transcriptomic profiles serve as a reliable surrogate for assessing real-time microbial functional dynamics, particularly in the absence of protein-level data. Future integration of complementary methods such as metaproteomics could further refine functional interpretations, but the current findings already provide meaningful and biologically relevant insights.* ***We also acknowledge the absence of healthy controls and extraction-negative controls in the current design. However, our objective was to compare disease-specific microbiome and resistome profiles between two infectious diseases. We plan to incorporate both extraction blanks and dengue-negative febrile illness controls in future work that will expand on this study by exploring severe and non-severe dengue cases.*** *Finally, all patient samples in this study were collected from North India, which may limit the generalizability of our findings to other geographic regions with differing healthcare infrastructure and antibiotic usage patterns. Moreover, while this meta-transcriptomic approach offers valuable insights, its broader adoption in public health surveillance remains constrained by cost, complexity, and infrastructure—particularly in low- and middle-income countries (LMICs). Streamlining protocols and developing targeted transcriptomic panels may help increase feasibility for routine surveillance and outbreak preparedness. Further research is needed to unravel these complexities and their implications for AMR management in viral infections.*”

We do recognize the value of including healthy or disease-negative controls, and in our next planned study, we intend to systematically include negative controls for extraction kits as well as dengue-negative febrile controls, alongside non-severe and severe dengue patients. Additionally, to address Reviewer’s comment, we performed analysis within the Dengue cohort, using an independent lab generated dataset. Towards this, we included a dengue-negative control group comprising 20 febrile patients who tested NS1 antigen-negative, and whose blood total RNA samples were processed and sequenced using the same protocol as for the 112 dengue-positive samples. Microbial taxa and antibiotic resistance genes derived from unmapped reads were compared between the groups, and statistical differences were assessed using PERMANOVA with 999 random permutations. Random permutations were controlled by stratifying the site origin type using strata argument within adonis2 function of vegan package in R, wherein we observed distinct clustering, suggesting that the observed TAMs and ARGs differences are attributable to the disease status.

a.	TAMs abundance (Dengue positive and Dengue negative patients)

b.	ARGs abundance (Dengue positive and Dengue negative patients)

**Figure:** PCoA Plots representing distinct abundance clustering of (a) TAMs and (b) ARGs between dengue-positive and dengue-negative samples.

**Part III – Minor Issues: Editorial and Data Presentation Modifications**
Reviewer #3: (No Response)

Best wishes,

**Rajesh Pandey, PhD**

Principal Scientist,

INtegrative GENomics of HOst-PathogEn (INGEN-HOPE) laboratory,

CSIR-Institute of Genomics and Integrative Biology (CSIR-IGIB),

North Campus, Near Jubilee Hall, Mall Road, Delhi-110007, India.

Contact: [rajeshp@igib.in](mailto:rajeshp@igib.in); [rajesh.p@igib.res.in](mailto:rajesh.p@igib.res.in); Tel.: 011-27002200 (Ext. 254)
